# COPS: A novel platform for multi-omic disease subtype discovery via robust multi-objective evaluation of clustering algorithms

**DOI:** 10.1371/journal.pcbi.1012275

**Published:** 2024-08-05

**Authors:** Teemu J. Rintala, Vittorio Fortino

**Affiliations:** Institute of Biomedicine, School of Medicine, University of Eastern Finland, Kuopio, Finland; Goethe University Frankfurt: Goethe-Universitat Frankfurt am Main, GERMANY

## Abstract

Recent research on multi-view clustering algorithms for complex disease subtyping often overlooks aspects like clustering stability and critical assessment of prognostic relevance. Furthermore, current frameworks do not allow for a comparison between data-driven and pathway-driven clustering, highlighting a significant gap in the methodology. We present the COPS R-package, tailored for robust evaluation of single and multi-omics clustering results. COPS features advanced methods, including similarity networks, kernel-based approaches, dimensionality reduction, and pathway knowledge integration. Some of these methods are not accessible through R, and some correspond to new approaches proposed with COPS. Our framework was rigorously applied to multi-omics data across seven cancer types, including breast, prostate, and lung, utilizing mRNA, CNV, miRNA, and DNA methylation data. Unlike previous studies, our approach contrasts data- and knowledge-driven multi-view clustering methods and incorporates cross-fold validation for robustness. Clustering outcomes were assessed using the ARI score, survival analysis via Cox regression models including relevant covariates, and the stability of the results. While survival analysis and gold-standard agreement are standard metrics, they vary considerably across methods and datasets. Therefore, it is essential to assess multi-view clustering methods using multiple criteria, from cluster stability to prognostic relevance, and to provide ways of comparing these metrics simultaneously to select the optimal approach for disease subtype discovery in novel datasets. Emphasizing multi-objective evaluation, we applied the Pareto efficiency concept to gauge the equilibrium of evaluation metrics in each cancer case-study. Affinity Network Fusion, Integrative Non-negative Matrix Factorization, and Multiple Kernel K-Means with linear or Pathway Induced Kernels were the most stable and effective in discerning groups with significantly different survival outcomes in several case studies.

## Author summary

We developed COPS (Clustering algorithms for Omics-driven Patient Stratification), a computational platform to tackle challenges in disease subtype discovery using single or multi-omics data. COPS employs innovative methods for assessing clustering stability and utilizes the Pareto optimal criterion to find solutions balancing clustering evaluation metrics and clinical relevance. Our comprehensive comparison across several multi-omic cancer datasets demonstrated the efficacy of both pathway-based and non-pathway-based methods in different contexts. Furthermore, we introduced two new methods utilizing pathway graph kernels and multiple kernel learning for pathway-based patient stratification. In our benchmarking study, we observed that different clustering algorithms yielded solutions with different trade-offs between clustering stability, association with known subtypes, and significance of survival differences between the clusters. This work underscores the need for future clustering algorithms to simultaneously address both stability and clinical relevance.

## Introduction

Clustering algorithms are often used in omics studies to identify groups of patients sharing similar molecular patterns. The underlying assumption is that these patient subgroups may represent novel disease subtypes with possibly different survival outcomes or differential response to various treatments [1,2]. Advancements in high-throughput sequencing has enabled clustering analysis across multiple omics layers like gene-expression, DNA-methylation, and somatic mutations to define disease subtypes. However, the high dimensionality of omics data makes generating relevant patient groupings extremely challenging for heterogeneous diseases like cancer. Popular methods often adopt an entirely data-driven approach, which, while important, could be improved by incorporating pathway information, particularly in fields like cancer biology. This integration may result in more interpretable clustering outcomes with enhanced prognostic relevance. However, although many pathway-based multi-view clustering methods have emerged recently, they have yet to be systematically compared with their data-driven counterparts. Moreover, current benchmarking studies do not provide a computational platform for testing and comparing multi-view clustering methods on novel datasets, allowing for the selection of the best clustering methods based on stability and other relevant metrics, nor do they permit access to both existing and novel pathway-based methods, thereby highlighting a significant gap in the methodology. Furthermore, these studies lack a multi-objective approach that could effectively evaluate which methods offer the best trade-off between clustering metrics.

Here, we present COPS (Clustering algorithms for Omics-driven Patient Stratification and robust evaluation of clustering results) an R package that provides quick access to a unique compendium of state-of-the-art clustering methods for single- and multi-omic data as well as a robust pipeline to evaluate the reproducibility and prognostic value of clustering results in addition to standard clustering metrics. S1 Fig provides a visual representation of the computational framework proposed for subtyping omics profiles, whether in a single- or multi-omics context. Within multi-omics methods, the most promising are so called intermediate integration methods which aim to analyze multi-view data in a holistic manner rather than analyzing each view separately or concatenating all features before applying machine learning. These methods, encompassing joint Dimensionality Reduction (jDR) techniques as well as similarity network fusion algorithms followed by clustering analysis, have been reviewed extensively over the past few years without a clear winner emerging [2–9]. However, an important category of algorithms, those founded on pathway- or network-driven approaches, has been absent from previous reviews. The integration of biological knowledge such as gene-networks and pathway gene sets with omics data has been underutilized in multi-omics-driven patient stratification. Several methods to integrate biological knowledge into multi-omics integration have been developed while only few have been reviewed [10]. Significantly, COPS stands out by offering several pathway-based, single- and multi-omics clustering methods. These methods are rooted in the core principle of transforming omics-level features into pathway or network-based information. For multi-omics, COPS offers the implementation of existing and novel algorithms that combine patient omics profiles with pre-existing biological knowledge networks. These methodologies leverage a graph kernel approach after mapping each omic data layer onto genes, and subsequently onto pathways [11,12]. We also implemented a novel pathway kernel that utilizes betweenness node centrality in pathway graphs to weight features when computing patient similarities, which yielded higher clustering stability compared to other similar methods. We also define a variant that uses the Random Walk with Restart algorithm to assess whether two patients have molecular feature alterations in close proximity within a given pathway, similar to the approach described in [12]. Kernel methods are in general an appealing choice since they are efficient for high-dimensional data with small sample sizes and they allow the separation of knowledge integration and machine learning algorithms to different steps. Moreover, within COPS, we also provide a variant of the spectral clustering algorithm which uses a QR-decomposition based algorithm [13] instead of k-means to potentially improve stability of previous pathway kernel clustering approaches. S1 Table comprehensively enumerates the full range of methods and functionalities offered by COPS.

The present study aims, however, to demonstrate the effectiveness of COPS in benchmarking multi-view clustering methods to categorize cancer patients based on multi-omics profiles. It also intends to evaluates our two new pathway graph kernels, respectively termed BWK and RWR-BWK, which may be better suited for clustering analysis than previous pathway graph kernels. While previous research has used metrics like ARI or F1-score to assess clustering accuracy, and survival analysis with the log-rank tests between clusters or proportional-hazards regression models using latent variables to assess the prognostic significance of cancer subtypes, they often overlooked critical factors. Notably, many comparative analyses of multi-view clustering in cancer research did not adjust survival analyses for covariates like age or cancer stage, which limits the relevance of the predictions. Moreover, the importance of subsampling in evaluating clustering metrics and stability has not been sufficiently explored in the multi-omic context. COPS addresses these gaps by integrating repeated subsampling assessments of clustering metrics, such as ARI, survival, and stability, and by considering trade-offs between them. It uses the Pareto optimal criterion to find all clustering solutions that optimally balance different metrics, highlighting solutions where (by selecting a different method or number of clusters) no one objective can be improved without expending another. This holistic approach provides a more robust comparison of multi-view clustering algorithms.

## Results

### Enhancing multi-view clustering algorithm benchmarks with COPS

**Fig 1:**
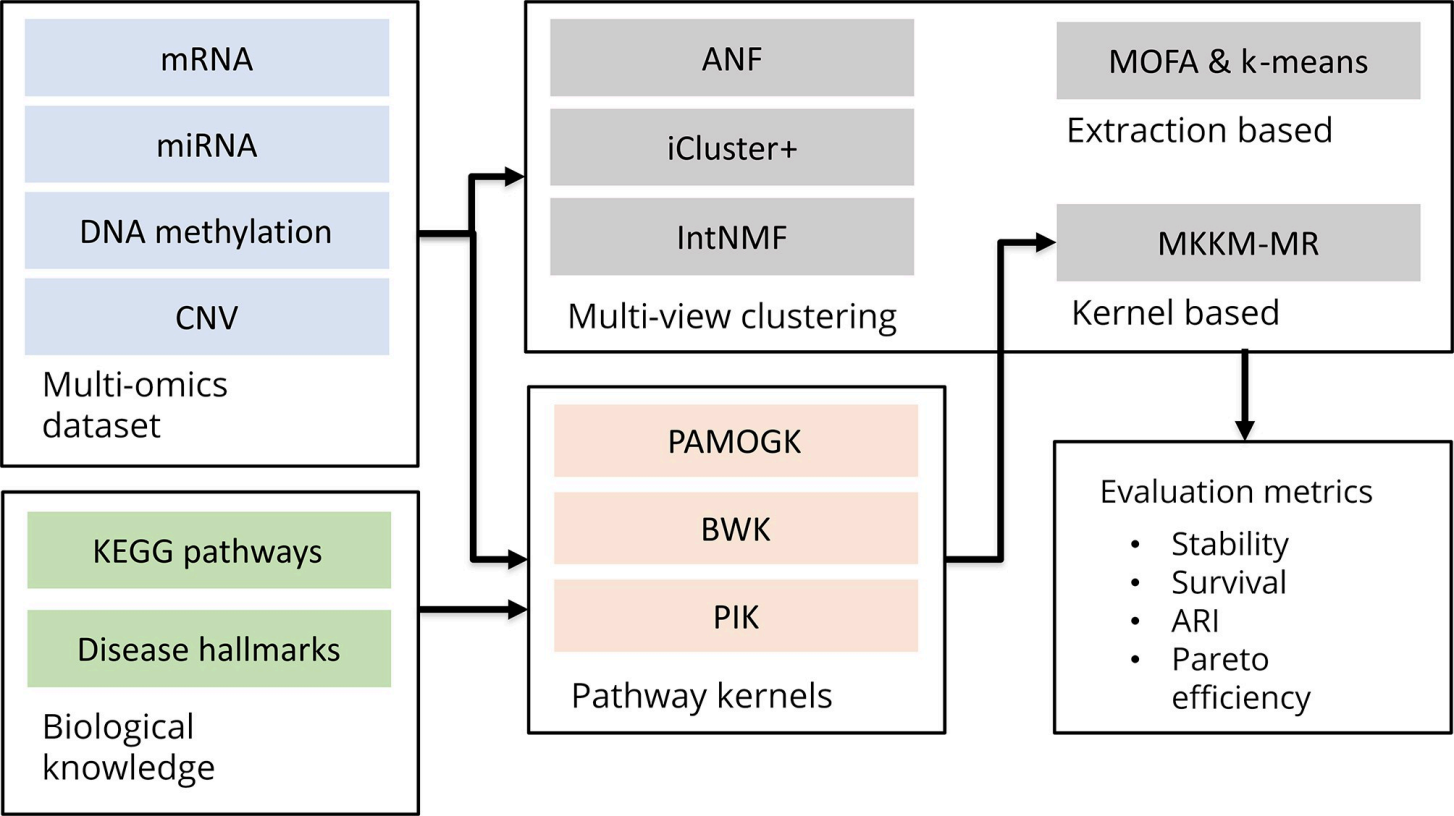
COPS benchmark workflow. Starting from multi-omic data, ANF, iCluster+ and IntNMF yield clustering results directly, MOFA is used to acquire lower dimensional representation that is clustered with standard algorithms, while MKKM-MR is used to fuse kernels before applying kernel k-means. For pathway-based kernels, cancer-associated KEGG pathways were used to define multiple pathway-kernels for each omic. Finally, clusters were evaluated with respect to clustering stability, survival relevance, and accuracy, as well as their trade-offs.

COPS was employed to identify subtypes for seven types of cancer, namely kidney, breast, prostate, thyroid, ovarian, lung, and brain cancer, using four different types of omics data: mRNA, miRNA, DNA methylation, and copy-number variants. Fig 1 visually represents the comparative studies conducted, while Table 1 consolidates the number of omics profiles, selected gold-standard subtypes, and the type of survival assessed for each benchmarked cancer dataset.

**Table 1:** List of benchmarking dataset from TCGA and the gold-standard subtype as well as survival type considered (OS–overall survival; PFI—Progression-free interval).

Cancer type	Samples	Subtypes	Survival	Reference
Breast cancer (BRCA)	727	PAM50 subtypes: 130 Basal, 44 Her2, 399 LumA, 128 LumB, 33 Normal-like, 9 missing	95 events (OS), 648 event-free	[20]
Renal cell carcinoma (RCC)	523	Histological subtypes: 183 clear cell, 274 papillary and 66 chromophobe	98 events (OS), 425 event-free	[21]
Low-grade glioma (LGG)	512	IDH mutation and 1p/19q co-deletion status: 169 IDHmut-codel, 245 IDHmut-non-codel, 95 IDHwt, 3 missing	124 events (OS), 387 event-free	[22]
Non-small cell lung cancer	788	Histological subtypes: 447 adenocarcinomas, 341 squamous-cell carcinomas	299 events (OS), 489 event-free	[23,24]
High-grade serous ovarian cystadenocarcinoma	286	Expression subtype: 64 Differentiated, 77 Immunoreactive, 70 Mesenchymal, 75 Proliferative	171 events (OS), 115 event-free	[25]
Prostate adenocarcinoma (PRAD)	493	ERG fusion status: 151 fusion, 179 no fusion, 163 missing	92 events (PFI), 401 event-free	[26]
Papillary thyroid carcinoma	500	BRAF-RAS scoring: 271 BRAF-like, 119 RAS-like, 103 unlabeled	51 events (PFI), 449 event-free	[27]

Additionally, S2 Fig provides a graphical illustration of the cross-validation based subsampling framework. Samples with missing or ambiguous labels (e.g., “other”) were omitted from gold-standard agreement evaluation but were included in the clustering analysis. To evaluate the performance of different clustering algorithms, we selected nine methods from COPS, including two joint dimensionality reduction methods (MOFA and integrative non-matrix factorization) [14,15], a network-based method (Affinity Network Fusion) [16], a statistical-based method (iCluster+) [17], Multiple Kernel K-Means with Matrix induced Regularization (MKKM-MR) paired with linear kernels and four different pathway graph kernel based methods including Pathway Induced Kernel (PIK) [11], PAthway MultiOmic Graph Kernel (PAMOGK) [12] as well as a novel Betweenness Weighted Kernel (BWK) and its variant RWR-BWK which applies an additional random-walk-with-restart procedure. These graph kernels utilize known biological pathways that were extracted from KEGG [18] or the National Cancer Institute Nature Pathway Interaction Database [19]. The proposed benchmark aims to evaluate the trade-offs between the statistical significance of survival analysis and the stability of clustering results for different numbers of clusters k, thereby simulating a discovery process for novel cancer subtypes. As a further evaluation strategy, we compare the clustering accuracies while using the number of gold-standard subtypes for k. Regarding hyperparameter tuning, we followed the precedent set by similar studies by using the default parameters of the available methods [7,9]. However, to reduce feature count for data-driven methods, we systematically applied a high-variance gene filter, a recommended feature selection step for factorization-based methods like iCluster+ and MOFA2. The pre-processing steps including the mapping of omics features to genes and the subsequent filtering procedure are detailed in the Materials and Methods section.

### Assessing cluster stability via repeated cross-validation

**Fig 2:**
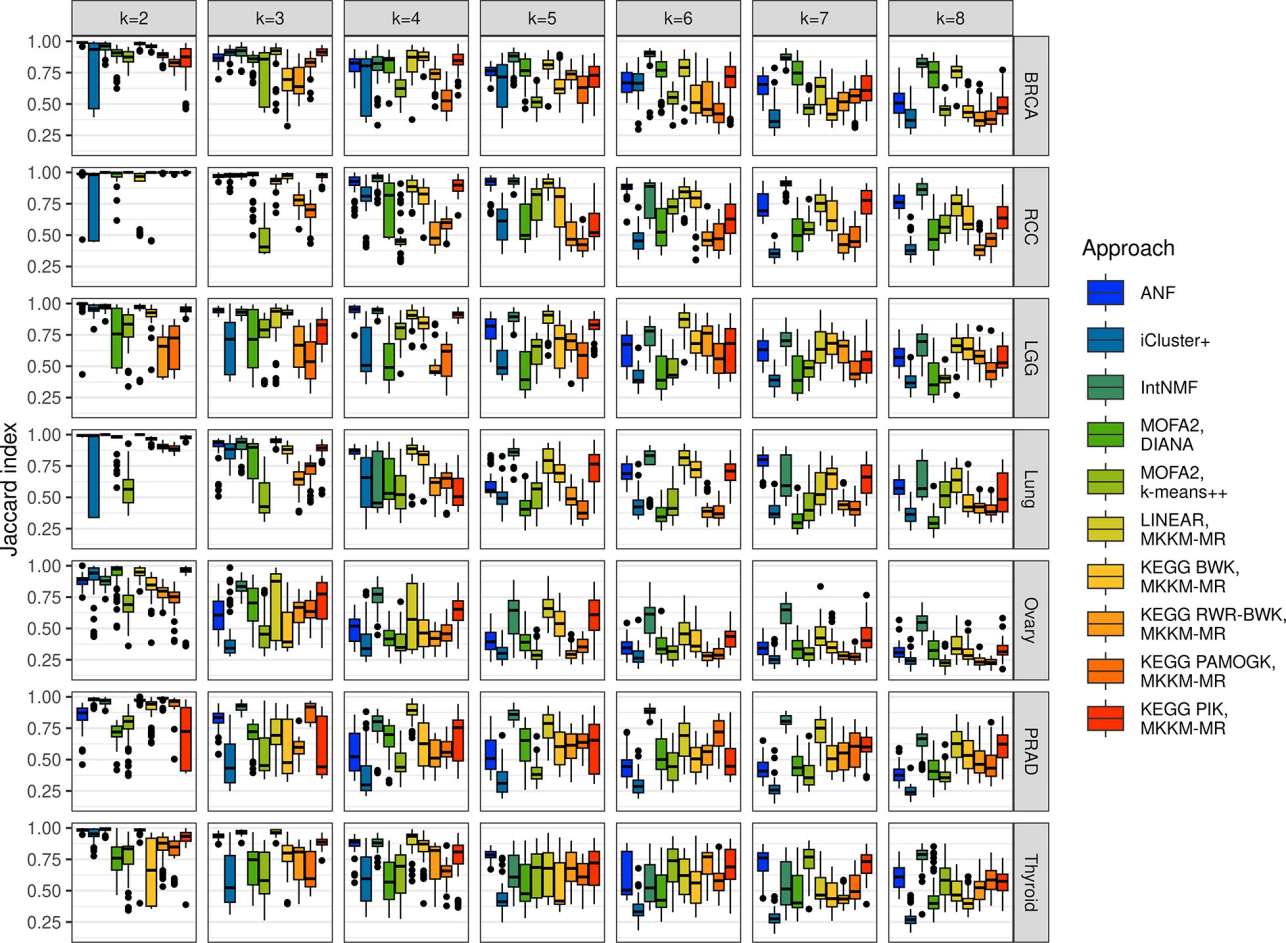
Stability of cancer clustering results. The boxplots show Jaccard-index-based stability for different multi-omics clustering approaches and different number of clusters (k) across 10 repeats of 5-fold cross-validation. The box and middle line represent the second and third quartiles and the median while the whiskers extend to the maximum value or 1.5 times inter-quartile range from the box edges.

Previous benchmarking studies have primarily focused on the accuracy of clustering algorithms and the significance of survival differences between groups of patients based on one-shot applications of the clustering algorithms. In contrast, we evaluated the stability and robustness of clustering results by subsampling real datasets multiple times. COPS is designed to evaluate the performance of clustering methods by simulating small differences in input data this way. Fig 2 compares the Jaccard index for partitions [28] across ten repeats of the 5-fold cross-validation process to assess the stability of clustering results as the number of clusters is set between 2 and 8. More details on the proposed repeated cross-validation and the formulation of the Jaccard index for clustering stability, which avoids the problem of cluster alignment, are provided in the Materials and Methods section.

Regarding the data-driven methods, the selection of the top M high-variance genes can impact the results in some case studies, as shown in S3 Fig. Therefore, the optimal M value, chosen from options of 500, 1000, or 2000 genes, was determined for each method and case study based on the average stability performances across k (number of clusters). Fig 2 displays only the best performances of M for each data-driven method and case study, while S2 Table indicates the M values selected for each case study and method.

Overall, the number of clusters impacts stability and different approaches yielded different numbers of stable clusters. Notably, results for two clusters were stable and tended to decrease with higher numbers of clusters. However, in some cases and with certain methods, stability increased again when considering a larger number of clusters. ICluster+ stability at two clusters was highly variable in three out of seven datasets, but in those cases the three cluster results were consistently stable and decreased afterwards, showing a clear peak stability at either two or three clusters. Among the knowledge integration methods, PIK and BWK exhibited similar performances and often outperformed both PAMOGK and RWR-BWK, approaching the performances of the best data-driven methods. However, when considering NCI-PID pathways (S4 Fig), PAMOGK exhibits higher stability in LGG, lung cancer, and ovarian cancer. PAMOGK and RWR-BWK showed lower stability scores beyond 2 clusters in most cases while BWK performed better, which suggests that the instability is at least in part caused by the random walk process. Notably, linear kernels tended to be more stable than pathway kernels, but this is to be expected since the number of kernels integrated is much higher for the latter. ANF and IntNMF stability was consistently high up to four or five clusters except for ovarian cancer and PRAD where ANF was stable only up to 2 and 3 clusters respectively. Meanwhile MOFA stability depended on the clustering method applied after factorization but in general was lower than other data-driven methods. To select the optimal number of clusters, the elbow method can be applied to stability, however, it is crucial to consider additional evaluation metrics alongside stability, as these may suggest different optimal numbers of clusters. Balancing these metrics can guide a more comprehensive and accurate determination of cluster count.

### Assessing clustering performance through survival data

**Fig 3:**
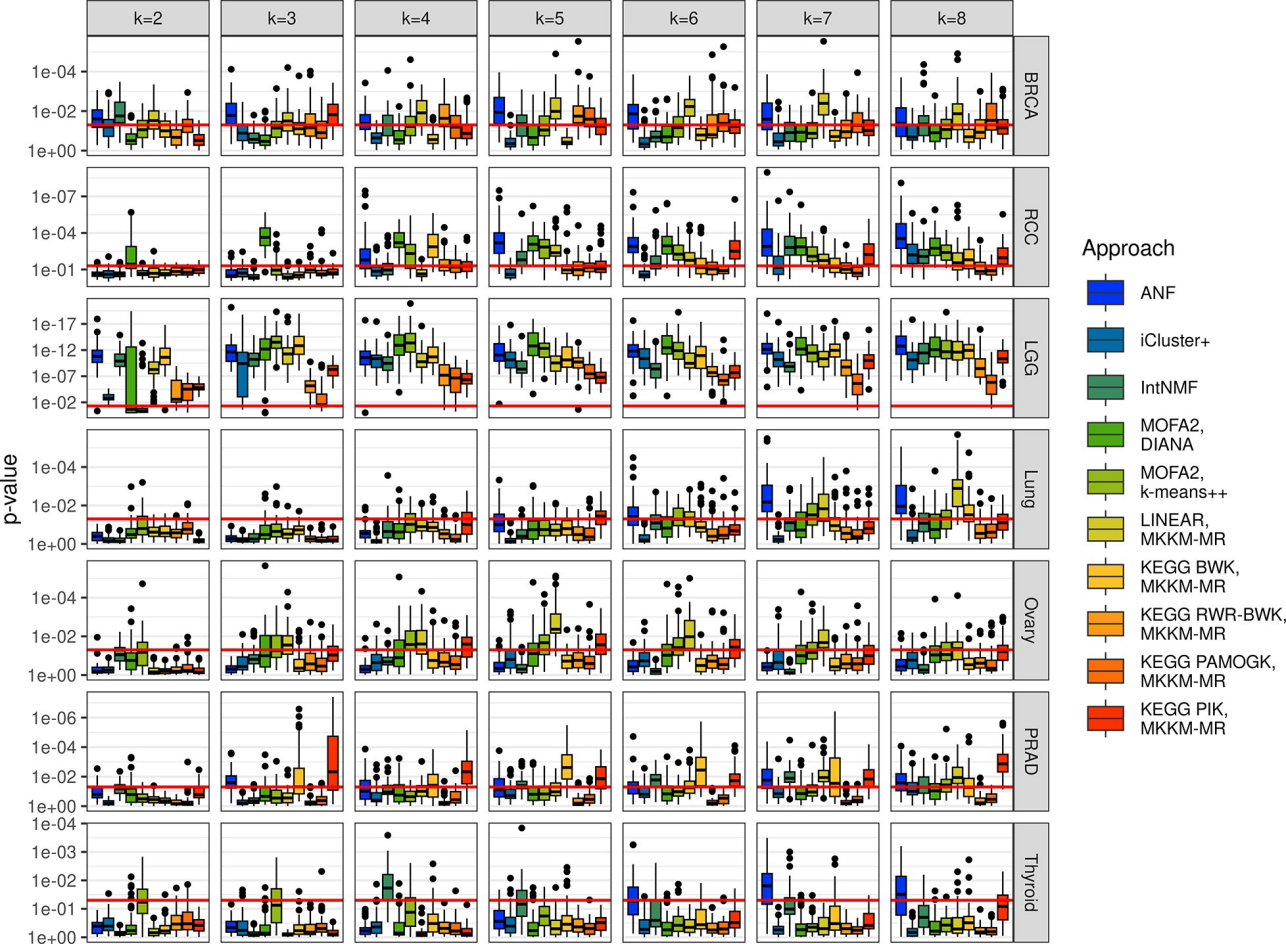
Cluster survival results. The boxplots show the p-value of a likelihood-ratio test between Cox PH models such that known covariates are accounted for. Values for different multi-omics clustering approaches and different number of clusters (k) across 10 repeats of 5-fold cross-validation. The box and middle line represent the second and third quartiles and the median while the whiskers extend to the maximum value, or 1.5 times inter-quartile range from the box edges. The red line represents the threshold p = 0.05.

We conducted survival analysis to assess the prognostic relevance of the clustering results. Differently from previous comparative studies, our survival analysis was based on the capacity of multi-view clustering algorithms to yield groupings that provide additional predictive value beyond clinically available information. Moreover, our findings provide insights into the potential of multi-omics clustering analysis for cancer subtyping and may have implications for improving patient stratification and personalized treatment. Fig 3 includes the p-values derived from the likelihood-ratio test between a Cox Proportional Hazards (PH) model using only clinical variables as predictors, and a model using the grouping as an additional predictor. The null hypothesis of this test is that the coefficients of group indicators are zero.

Survival relevance varied significantly across different cancer types: Most algorithms consistently identified relevant groupings in RCC and LGG, whereas in lung, ovary, prostate, and thyroid cancer most algorithms failed to generate significant groups at any number of clusters. Among the algorithms, ANF and linear MKKM-MR generally delivered good performance across most cancer datasets, typically achieving significance with higher numbers of clusters. When considering pathway-based methods, BWK followed by PIK appears to outperform other pathway-based methods. Moreover, in prostate cancer, PIK and BWK have outperformed data-driven methods and achieved significant results in RCC and LGG. Notably, BWK’s performance in LGG is comparable to that of the best data-driven methods. PAMOGK and BWK-RWR deliver their best relative performance in terms of survival relevance in BRCA, where they outperform other pathway kernels. Excluding the LGG case study, prognostic relevance tended to increase on average with higher numbers of clusters.

This finding reveals a significant trade-off between stability and prognostic relevance, underscoring the importance of utilizing multiple evaluation metrics and visualization tools to balance these conflicting objectives effectively. We also analyzed prognostic relevance for the pathway-based methods using the NCI-PID and the results were included as S5 Fig, where they are compared with KEGG pathway results.

### Evaluating the association of clustering results with known patient subgroups

**Fig 4:**
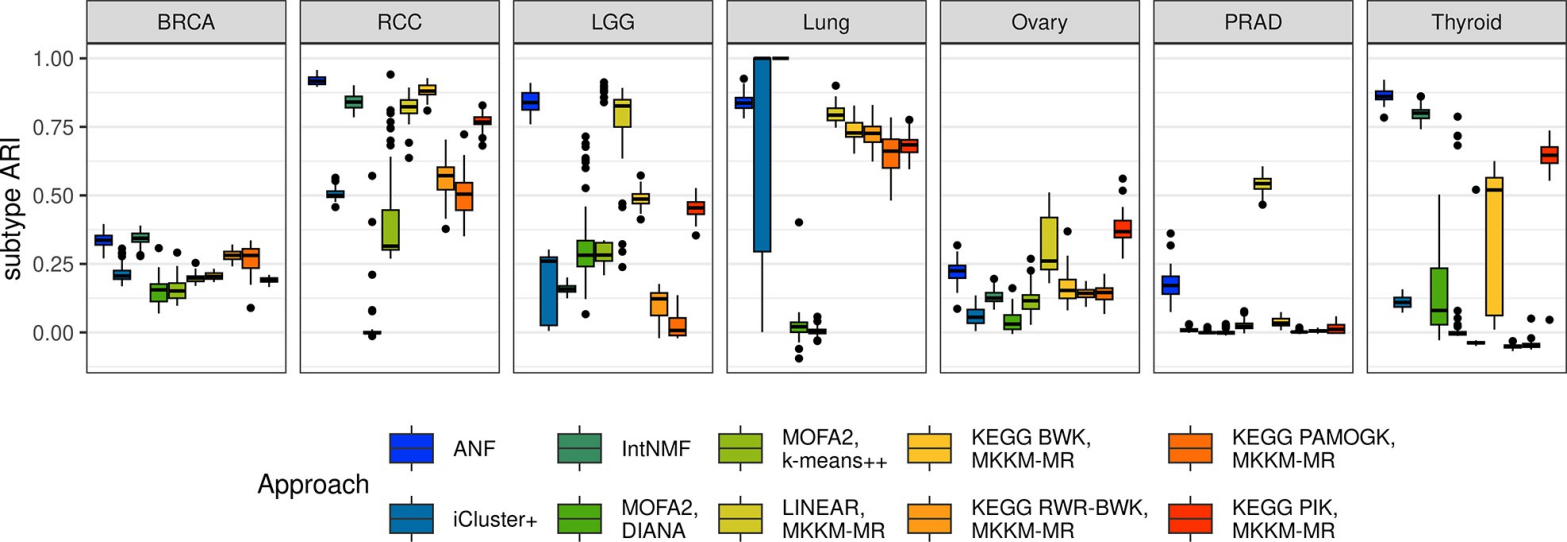
Cancer subtype agreement. The boxplots show the adjusted rand index (ARI) between the clusters and gold-standard subtypes on the y-axis. For each dataset only the clustering result corresponding to the known number of subtypes is shown for the considered multi-omics clustering approaches across 10 repeats of 5-fold cross-validation. The box and middle line represent the second and third quartiles and the median while the whiskers extend to the maximum value or 1.5 times inter-quartile range from the box edges.

The association between patient grouping results and known molecular or histological cancer subtypes was assessed using the Adjusted Rand Index (ARI) [29]. To identify suitable gold-standard subtypes, we applied two different strategies: either the datasets were combined from multiple major histological subtypes, as in the case for kidney and lung cancer, or we used the molecular subtypes with strong evidence in the original TCGA publications (see Table 1). Fig 4 presents the ARI scores achieved through repeated cross-fold validation for the correct number of clusters, i.e. the number of gold-standard subtypes. In our analysis of BRCA, we observed that most methods achieved an ARI between 0.2 and 0.4, reflecting prior findings [9].

The RCC dataset presented strong performance and low variance with ANF, IntNMF as well as the linear kernels, BWK and PIK. In LGG, ANF and the linear kernel were the highest performing methods with median ARIs of 0.84 and 0.83 respectively. In the non-small cell lung cancer dataset IntNMF had perfect accuracy in distinguishing the major histological subtypes. ICluster+ also had perfect accuracy more than half of the time, but the other portion of results had very poor accuracy yielding drastically different results in some subsamples of the data. ANF and all kernel methods also yielded high ARI scores, and only MOFA2 yielded clusters without any association to the subtypes. For the ovarian cancer dataset, we examined subtypes characterized based on mRNA. Based on our results, these subtypes were not recapitulated using a multi-omics approach, although, the linear kernel and PIK reached moderate agreement similar to the best results for BRCA subtypes which were also determined from gene-expression. In prostate cancer, only the linear kernel performed well (median ARI of 0.54) with the correct number of clusters (2 for ERG fusion or no fusion), but ANF and IntNMF also achieved decent performance with median ARIs of 0.39 and 0.33 respectively when three clusters were considered (S6 Fig). This may indicate that there is another molecular pattern that drives most clustering algorithms more strongly than the ERG fusion. In thyroid cancer, ANF and IntNMF were again performing significantly better than other methods and achieved median ARIs of 0.86 and 0.80, respectively while PIK ARI was 0.65. It should be noted that PAMOGK achieves slightly higher ARI scores when considering NCI-PID pathways (S7 Fig).

The ARI has been frequently employed as a principal clustering evaluation metric. However, in cancer datasets, subtypes derived from one type of molecular data often do not align with subtypes derived from other types of molecular data. Despite our efforts to identify the most relevant subtypes, our selection may not necessarily represent the full molecular variation of the combined omics data types. For instance, the thyroid selected subtype was based on two mutually exclusive driver mutations with highly impactful signaling pathway effects [27] that were recovered quite well with the most reliable methods in our tests while the breast and ovarian cancer subtypes were based on mRNA expression clustering into five and four clusters with biological relevance [25,30–32], which makes them more difficult to reproduce exactly even when using gene-expression data alone. On the other hand, the histological subtypes in RCC and lung cancer have intrinsic differences in their expression and methylation profiles resulting in easily distinguishable patterns. Regardless, studies concerned with novel subtype discovery are better served by considering multiple approaches and robust assessment of stability and survival rather than selecting methods that can reproduce prior results.

## Exploring trade-offs in multi-omics-driven cancer subtyping using stability assessment and survival analysis

**Fig 5:**
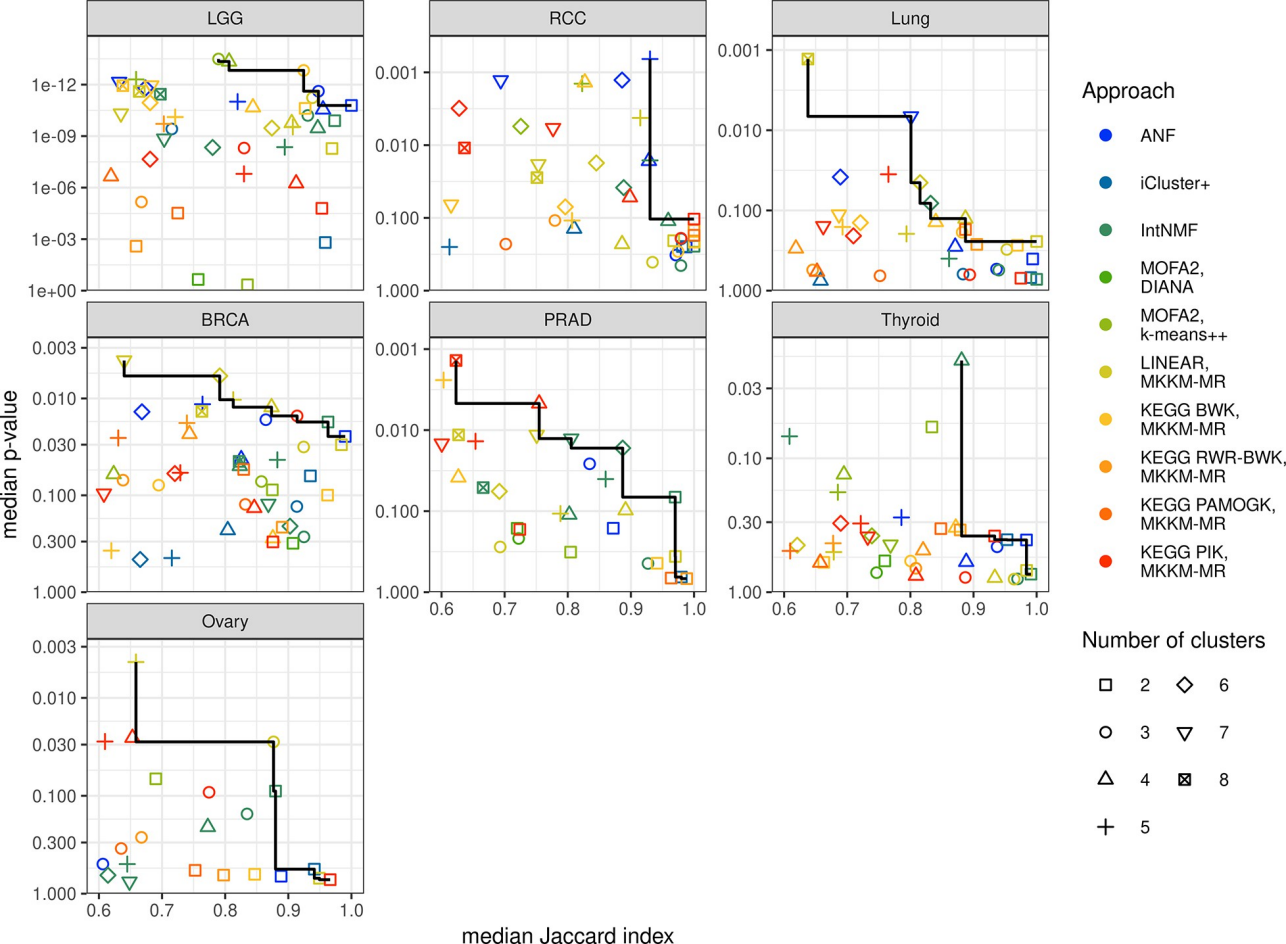
Multi-objective clustering result evaluation. Each point represents a clustering result obtained using different methods and numbers of clusters. The x and y axis represent the median survival p-value and clustering stability Jaccard index calculated across resampled datasets. The line connects the set of non-dominated results.

In this section we evaluate clustering results metrics using the Pareto criterion, which can help identify non-dominated solutions or clustering results that optimize multiple criteria simultaneously. In the previous sections, we noted that multi-omics patient stratification via clustering algorithms often yields conflicting performance outcomes across various cluster quantities within cancer datasets. Therefore, it is often not possible achieve optimal performance in all criteria simultaneously as improving one criterion may negatively impact another. The Pareto criterion provides a way to evaluate clustering results by identifying the set of non-dominated solutions that cannot be improved in one performance metric without negatively impacting any other metrics. By analyzing all non-dominated solutions, one can make informed decisions based on a comprehensive evaluation of multiple criteria, such as cluster stability and patient survival. This is particularly important in the context of cancer subtype discovery, where clustering results may have far-reaching consequences on patient diagnosis and treatment. Fig 5 presents the initial set of non-dominated solutions (the closest approximation of the Pareto optimal front) for each cancer type using the benchmarked multi-view clustering algorithms. Clustering solutions with median smallest cluster size below 20 samples and solutions with median stability below 0.6 were omitted.

For LGG, ANF, BWK, and MOFA2 with k-means++ exhibited superior performance compared to other methods, although there was no consensus regarding the optimal number of clusters. In RCC, there was no significant trade-off between the relevance of survival and clustering stability as ANF showed an optimal compromise at 5 clusters with a median p-value below 0.0007 and median stability of 0.93 followed by the 6-cluster result that had close but slightly worse performance. This number is intriguing, given that the histological classification for the papillary subtype, can be divided into type 1 (pRCC1) and type 2 (pRCC2) [33]. While pRCC1 tumors tend to be smaller and less aggressive, pRCC2 tumors are often larger and more aggressive. Furthermore, recent studies highlight the molecular-level heterogeneity of the clear cell carcinoma subtype (ccRCC), which could potentially be segmented into 2 to 4 clusters [34]. This indicates that using analyses like those incorporated in COPS could potentially enable a more accurate determination of the appropriate number of clusters. In lung cancer, the non-dominated set contained results from ANF, IntNMF, linear kernel, and PAMOGK, however, it could be argued that the best compromise was given by ANF at seven clusters with median stability of 0.80 and survival p-value below 0.007. Results with higher stability were not significant from the survival perspective and the more significant 8-cluster linear kernel result was quite unstable at 0.64 Jaccard index. In BRCA, the set of non-dominated solutions included IntNMF, ANF, linear kernel, and PIK, where many 2-cluster results achieved survival significance at high stability and cluster number up to 6 were mostly stable with slightly higher survival significances. In PRAD, IntNMF and PIK offered the best trade-offs with varying numbers of clusters with IntNMF yielding higher stability and reaching survival significance at 6 clusters. In thyroid cancer IntNMF with 4 clusters was the only solution with median survival p-value below 0.05 and this solution was also highly stable making it the best method in this case study. In ovarian cancer, IntNMF, linear kernel, and PIK were in the non-dominated set, but the 3-cluster linear kernel was the only one to achieve both survival significance and high stability. It is noteworthy that almost all of the considered methods appear in the non-dominated set in at least one of the case studies. Out of the pathway-kernel based methods PIK and BWK demonstrated the most utility by providing competitive performance compared to data-driven methods in PRAD and LGG, respectively. Additionally, it should be noted that RWR-BWK and PAMOGK tended to yield more imbalanced clusters in terms of size, as demonstrated in S8 Fig, which reports the size of the smallest cluster. This imbalance made them less likely to be included in the Pareto fronts, especially considering their overall low stability.

## Discussion

In our study, we employed COPS for multi-omics clustering on seven cancer types, including breast, prostate, and lung, among others, using mRNA, CNV, miRNA, and DNA methylation data. Unlike prior studies, we compared data- and knowledge-driven multi-view clustering methods, while also incorporating subsampling to ensure robust assessment of results and metrics. We focused on kernel-based knowledge driven clustering methods due to their performance advantage in analyzing high-dimensional data and the ability to separate the kernel from the algorithm which enabled us to compare the same clustering algorithm between data- and knowledge-driven kernels and to use PIK which was originally developed for a supervised learning task. We also implemented two novel knowledge-driven approaches that utilize the concept of node betweenness in pathway networks. These were compared agains other pathway-driven, multi-view kernel clustering approaches. Clustering outcomes were evaluated based on three key metrics: the ARI score, survival analysis conducted through Cox regression models accounting for relevant covariates, and the stability of the clustering results. Subsequently, Pareto efficiency was analyzed to determine the balance of evaluation metrics and to identify superior algorithms based on their proficiency in recognizing solutions on the initial Pareto frontier. The stability analysis highlighted that many algorithms, such as ANF, IntNMF, and MKKM-MR with linear kernels, BWK, or PIK, were effective in identifying stable clusters while iCluster+, MOFA2, and MKKM-MR with RWR-BWK or PAMOGK were less stable, which suggests an elevated sensitivity to sampling variability. ANF, IntNMF, and MKKM-MR with linear or pathway induced kernels frequently excelled in identifying both stable and prognostically significant patient groups. Furthermore, other methods, such as BWK MKKM-MR, yielded relevant results in specific datasets by providing unique trade-offs between stability and survival. Out of the pathway based methods BWK and PIK seemed most promising, however, it should be noted that combining these methods with different pathway interaction databases such as Reactome [35] or the National Cancer Institute Pathway interaction database [19], could yield different results. Selecting a limited set of key pathways specific to the disease is recommended since each pathway-omic combination is represented by a different kernel and a large number of noisy kernels can overwhelm the integration algorithm. For this reason, we selected a cancer-associated subset of 48 KEGG pathways for the main figures, but also tested the NCI-PID pathways of which 210 included overlapping genes with the TCGA datasets (See S4, S5, and S7 Figs). The PID results for RWR-BWK and PAMOGK showed higher stability at the expense of survival significance while BWK and PIK achieved slightly higher survival significance in RCC and PRAD respectively. These results were aligned with our observations of the best methods in the KEGG results suggesting that the PID networks were not too noisy or numerous although the required computational resources were higher. The Reactome database is considerably larger than either KEGG or NCI-PID, hence we did not include it in our testing.

Our findings emphasize the need for careful evaluation of multi-view clustering methods. While survival analysis and gold-standard agreement are popular evaluation metrics, they can exhibit high variance in some methods. While it is important to assess whether the subtypes identified by clustering methods are associated with established cancer subtypes, this evaluation should not necessarily guide the selection of the overall best method. As the full interplay between different omics in the pathogenesis of cancer is still not fully understood, it would be unreasonable to assume that current subtypes represent the expected clustering result of a perfect algorithm. Moreover, the results with highest survival differences were attained with a number of clusters much higher than the number of histological subtypes which highlights the need for further subdivision. Furthermore, different methods offered different trade-offs in each dataset. Therefore, it is crucial to test different clustering methods and evaluate a variety of criteria, including survival relevance and clustering stability to select the most appropriate solution for the specific research question and dataset at hand.

## Materials and methods

### COPS—Clustering algorithms for Omics-driven Patient Stratification and robust evaluation of clustering results

The COPS R-package is available at https://github.com/UEFBiomedicalInformaticsLab/COPS. The repository includes three vignettes that cover an introduction to the COPS package, a multi-omics case study using the TCGA-BRCA multi-omics dataset, and a multi-cohort RNA-Seq dataset of patients with psoriasis. This psoriasis dataset is publicly available on a Zenodo repository [36]. While other R-packages provide access to a plethora of internal metrics to evaluate clustering results, COPS provides a few unique functionalities for omics data in life sciences. First, COPS uses repeated cross-fold validation to assess the reproducibility and stability of clusters and metrics on subsets of the data. Second, COPS offers access to both data-driven and biological-driven methods for single and multi-omic clustering analysis. In addition to clustering, biological-driven methods enable intermediate integration by transforming the data into pathway or network-based representations. Third, COPS provides robust test for survival differences between clusters while accounting for relevant covariates. Fourth, the COPS pipeline can be run for different combinations of feature-extraction and clustering algorithms. This includes standard clustering algorithms, dimensionality reduction methods and pathway enrichment methods from existing R-packages while providing a framework for evaluating different combinations of methods. In addition to readily available methods, we implemented several emerging methods which were not accessible through official R-packages such as 1) community detection on k-nearest-neighbour graphs [37]; 2) two multi-view kernel methods including Multiple Kernel K-Means with Matrix induced Regularization [38], and Multi-view Clustering with Enhanced Consensus [39]; 3) pathway based kernels from [11,12]; 4) the DiffRank pathway-activity inference method [40]; 5) two novel pathway kernels weighting features based on pathway network betweenness-centrality; 6) a novel network-based pathway enrichment analysis method combining of Random Walk with Restart and Fast Gene Set Enrichment Analysis (RWR-FGSEA) proposed by us [1]. We aimed to include multiple methods for unsupervised integration of prior biological knowledge, since we have shown that utilising these methods can improve the prognostic relevance of the clusters [1]. Table 2 summarises the different methods and the integrated knowledge input where appropriate.

**Table 2:** List of methods available in COPS grouped by type of method and the required inputs which correspond to prior biological knowledge that is integrated. Different feature extraction and clustering algorithms can be used in multiple configurations.

Type	Methods	Integrated knowledge
Pathway gene-set based feature extraction	DiffRank, GSVA	Feature-sets corresponding to pathways, e.g., KEGG pathway genes
Gene-network based pathway feature extraction	RWR-FGSEA	One feature-network, e.g., gene-gene-network, pathway-feature sets
Pathway gene-network based feature extraction	Pathway Induced Kernel (PIK), Pathway Multi-Omic Graph Kernel (PAMOGK), Betweenness Weighted Kernel (BWK), RWR-BWK	Set of pathway feature-networks, e.g., KEGG pathway networks
Dimensionality reduction-based feature extraction	PCA, t-SNE, UMAP	None
Joint dimensionality reduction-based feature extraction	MOFA2	None
Single-view clustering methods	k-means++, agglomerative hierarchical clustering, DIANA, GMM, kNNG Louvain, spectral, kernel k-means	None
Multi-view clustering methods	ANF, iCluster+, iClusterBayes, IntNMF, MKKM-MR, ECMC	None

### Data collection and pre-processing

The omics data used for the benchmarking case studies were retrieved by using the curatedTCGAData R-package [41]. The retrieved data included upper-quartile normalized mRNA TPM based on RNA-Seq experiments quantified with RSEM [42], miRNA expression level, DNA methylation from Illumina BeadChips (27k and 450k) and somatic gene specific copy-number variations (CNV) which had been estimated from DNA sequencing data by using GISTIC2 [43]. The primary omics types used were mRNA, miRNA, and DNA methylation, however due to stability issues, miRNA in the breast cancer dataset was replaced with CNVs which improved the clustering stability of most methods. RNA-Seq data was log-transformed with pseudo count of 1. In order to improve performance of methods relying on Gaussian likelihoods, percentage methylation data was converted to M-values by using the logit-function [44], except for IntNMF inputs which must be non-negative. We used the 450k BeadChip data whenever it was available and only used 27k BeadChip data for the ovarian cancer where the 450k data was absent. Methylation probes from sex chromosomes were removed if data contained both sexes (i.e., in RCC, LGG, lung and thyroid datasets), in addition probes with more than 20% missing values were discarded and the remaining missing values were imputed using the k-nearest-neighbour method from the impute R-package with k = 10. In the TCGA breast cancer dataset we noticed outliers in the methylation profiles that were associated with the plate ID A161 which were removed from the dataset. Furthermore, miRNA and methylation data were summarized at gene level to reduce noise. MicroRNAs were mapped to genes by using miRNet [45] human miRNA-gene interactions database file “miRNet-mir-gene-hsa-tarbase.csv” (retrieved on September 3^rd^ 2022). The mean miRNA level was computed for all miRNAs interacting with each gene. Similarly, we used probe annotations from the IlluminaHumanMethylation450kanno.ilmn12.hg19 R-package to average the probe values associated with the promoter region of each gene. To achieve non-negativity for IntNMF in the CNV data, a pseudo-count of 2 was added since the loss of more than 2 copies of a gene is highly improbable and did not occur in the data. Most multi-omic integration methods require complete omics profiles, so samples missing any omic layer were omitted. Feature selection is highly recommended for factorization-based methods such as iCluster+ and MOFA2. For each method we selected the M highest variance genes from each omic and the union of them was used to select all matching features for clustering. M was selected between 500, 1000, and 2000 based on the average clustering stability of each method in each dataset. S4 Fig shows the stability for each M, k, approach and dataset. The feature selection step was omitted for pathway-based methods to maximize overlap with pathway genes to improve pathway integration.

### Selected multi-view clustering methods

The multi-view clustering methods considered include ANF [16], iCluster+ [17], IntNMF [15] and multiple kernel k-means with matrix induced regularization (MKKM-MR)[38]. Kernels based on integration of molecular pathways i.e., small networks of gene products, have been shown to improve prognostic relevance of clustering results in kidney renal clear cell carcinoma (KIRC) [12] and subtype classification in breast cancer [11]. We used R-package implementations when available and implemented the kernel methods in R with the MOSEK optimization toolkit [46]. Several of the tested approaches include hyper-parameters related to convergence and regularization which were set to default values in our testing. Hyper-parameter tuning can improve performance but typically the tuning process increases computation time and was also omitted in some of the previous comparisons [7,9].

#### Multi-view factor analysis

Multi-Omics Factor Analysis (MOFA) [14] is based on Bayesian factor analysis and can be used to factorize data from multiple views into a shared set of factors. These factors represent latent variables that can explain most of the variance in the data similarly to principal component analysis and they can be used to perform standard clustering analysis. MOFA was coupled DIANA [47] and k-means++ [48] to yield multi-omics clusters from the shared latent factors. Default settings were used with *convergence*_mode set to “medium”. MOFA uses regularization to select the optimal number of factors and the default maximum was 15 factors, which is sufficient for clustering analysis.

#### Multi-view clustering algorithms

Some of the most popular clustering algorithms for multi-omics data are also based on factorization but are specifically focused on grouping similar samples. The iCluster+ algorithm factorizes multi-view data into k-1 latent variables whose posterior means are used in k-means to yield the clustering result [17]. The IntNMF algorithm uses Non-negative Matrix Factorization to represent the data using k shared factors for all views which also determine the cluster membership [15]. The resulting factorization only contains non-negative values and can be interpreted as a mixture of components. ANF [16], on the other hand, utilizes affinity networks initially defined separately for each view and then integrated by using a network fusion algorithm. ANF uses spectral clustering to yield clustering results from the fused affinity networks.

#### Knowledge integrating kernels

The kernel represents the data via a kernel-function that defines the inner product between two input vectors. Knowledge integrating kernel-functions transform the data into a kernel matrix while utilizing prior knowledge of the relationships between the input features to capture patterns aligned with those relationships. The pathway induced kernel (PIK) uses the graph Laplacian of a pathway network to define the inner product for its kernel [11].

$$K_{L}(x,y)=x^{T}Ly,$$ (1)

where ℒ is the normalized Laplacian matrix of a given pathway network. According to the authors this kernel can be interpreted as diffusion potentials across pathway interactions. PIK was originally developed for a supervised learning problem, but here we have applied it in an unsupervised setting.

The PAthway-based Multi-Omic Graph Kernel (PAMOGK) [12] was developed for clustering omics data and the authors showed that it improved survival relevance of the clusters in their case-study. It uses pathway graphs in two different steps. Firstly, a random-walk-with-restart is used to smooth genetic features:

$$S_{g}^{\left(t+1\right)}=\alpha S_{g}^{\left(t\right)}A_{g}+(1-\alpha )S_{g}^{\left(0\right)},$$ (2)

where $S_{g}^{\left(t\right)}$ is the patient-gene matrix of smoothed features associated with pathway graph index *g* after *t* iterations of random walk, *α* is the complement of walk restart probability, *A*_*g*_ is the adjacency matrix associated with the graph *G*_*g*_, and $S_{g}^{\left(0\right)}$ is the initial matrix of patient pathway-graph node-associated features which are first standardized to 0 mean and unit variance and separated into two matrices based on the sign of the feature. Then the feature matrices may be binarized, thresholded or left as-is corresponding to Disc, TCont, and Cont in original paper. We used binarized features since clustering seemed to converge faster on average and in their original analysis binarized and thresholded features performed similarly well, which was also confirmed in our testing. Eq 2 is used to update $S_{g}^{\left(t\right)}$ until convergence. In the second step of PAMOGK kernel formulation, shortest path between each pair of nodes in each pathway graph is determined and an inner kernel is calculated for this path using the smoothened genetic features corresponding to the nodes constituting the path. Then the kernels are added together to form one kernel for each pathway, omic, and feature sign set. When the linear inner kernel is used, the PAMOGK kernel, Smoothed Shortest-Paths Kernel (SmSPK), is defined as:

$$K_{SmSPK}(G_{g}^{\left(i\right)},G_{g}^{\left(j\right)})=\sum_{p\in P_{g}}\langle s_{p}^{\left(i\right)},s_{p}^{\left(j\right)}\rangle =\sum_{p\in P_{g}}\sum_{v\in p}s_{g}^{\left(i\right)}(v)s_{g}^{\left(j\right)}(v),$$ (3)

where $G_{g}^{\left(i\right)}$ is the genetic feature attributed graph associated with sample *i* and graph index *g*, *P*_*g*_ is the set of shortest paths between all pairs of nodes in the pathway graph *G*_*g*_, $s_{p}^{\left(i\right)}$ is the vector of node attributes of $G_{g}^{\left(i\right)}$ along the path *p*, $s_{g}^{\left(i\right)}(v)$ retrieves the attribute value for *v*, and 〈∙,∙〉 is the dot product. Notably the PAMOGK implementation uses shortest paths including the end nodes and if there are multiple equal length shortest paths between two nodes only the first one discovered by a Breadth First Search is used which may change the result depending on the order of the network edges. The computational time is dominated by the inner kernel computation which involves a matrix multiplication between a *n*×|*p*| matrix and its transpose resulting in high complexity relative to standard kernels (e.g., linear or RBF) or the PIK. Furthermore, RWR may not be helpful for continuous genetic features such as gene-expression, since it smooths the original differences and binarizing or thresholding the z-score dichotomizes the feature space even if no large differences exist. To address these shortcomings, we implemented a different approach that makes use of topological characteristics of vertices in a pathway network instead.

The betweenness centrality of a node *v* is defined as:

$$B_{g}(v)=\sum_{i\neq j\neq v,j>i}\frac{\sigma_{ij}(v)}{\sigma_{ij}},$$ (4)

where *σ*_*ij*_ is the number of shortest paths between nodes *i* and *j* in graph *G*_*g*_, *σ*_*ij*_(*v*) is the number of such paths including *v*, and in case *i* and *j* belong to different disjoint components, i.e. no path exists between them, the corresponding term is considered 0 [49]. Note that Eq 4 excludes the nodes *i* and *j*, which often results in a value of 0 for peripheral nodes. Therefore, to avoid 0s we include the end nodes by defining $t_{i}=B_{g}(v_{i}){+r}_{i}\forall v_{i}\in G_{g}$, and $u_{i}=\sqrt{t_{i}}$, where *r*_*i*_ is the number of nodes reachable from *v*_*i*_. We then define a Betweenness Weighted Kernel (BWK) as:

$$K_{BWK}=\langle u{⊙x}_{g}^{\left(i\right)},u{⊙x}_{g}^{\left(j\right)}\rangle =\sum_{v\in V(G_{g})}u(v)x_{g}^{\left(i\right)}(v)x_{g}^{\left(j\right)}(v),$$ (5)

where ⊙ is the elementwise product between vectors. *K*_*BWK*_ is analogous to weighting each input feature with the betweenness centrality of the corresponding node in the pathway graph. If there are no multiple shortest paths between any nodes in the pathway graphs or if only the first path is used it is equal to the RHS of Eq 3. Therefore, by performing feature smoothing and calculating betweenness using first paths only *K*_*SmSPK*_ = *K*_*BWK*_. We consider the smoothing step optional and therefore define RWR-BWK as a variant that includes smoothing. In this article BWK and RWR-BWK refer to *K*_*BWK*_ with all shortest paths while PAMOGK refers to *K*_*BWK*_ with first shortest paths.

To define pathway graphs we retrieved KEGG pathways [18] by using the pathview R-package [50] and processed them to igraph-objects by using NetPathMiner R-package [51]. Different modes of interactions were ignored, and all edges were interpreted as undirected and unweighted. In general, any gene-interaction network and gene-set database could be used to define pathway sub-graphs, however, KEGG offers a relatively small number of high-quality pathways including the network topology. To further reduce the set of pathways we only used pathways associated with the hallmarks of cancer according to the *supplementary Table 1* reported in [52].

#### Multiple kernel clustering

An efficient multiple kernel learning (MKL) algorithm is crucial when using pathway-based kernels since each pathway is typically represented by a different kernel. A multiple kernel k-means algorithm based on matrix regularization (MKKM-MR) [53] was previously identified as a good performer in multi-omic clustering [12]. Hence, we used the MKKM-MR method to fuse the kernels and then applied spectral clustering [54]. All kernels were centered and normalized to unit L2-norm before applying MKL.

### Evaluation metrics for clustering analysis in cancer patient subtyping

The software tool COPS offers four kinds of clustering evaluation metrics, essential for evaluating cancer patient stratification results. These metrics are all assessed using cross-validation (CV), as shown in S3 Fig. COPS also captures metrics like the silhouette score and cluster associations with clinical variables. Table 3 summarizes metrics included in COPS. Besides offering a range of clustering metrics, the primary goal of COPS is to facilitate the comparison of clustering outcomes across multiple objectives using the Pareto front concept. When objectives are in conflict, metrics may show trade-offs. COPS aids in pinpointing optimal solutions by balancing these metrics, highlighting those meriting deeper exploration.

**Table 3:** Lists of metrics available in COPS, grouped by metric type and purpose for which they can be applied. Metrics reported in bolds were used in this study. The metrics highlighted in bold were employed in this study.

Type	Metrics	Purpose
**Internal quality indices**	E.g., **average silhouette width**, Calinski-Harabasz index, Davies-Bouldin index, Dunn index	Used to compare cluster separability and cohesion between methods
**Clustering stability**	Adjusted Rand Index**, Jaccard index** and normalized mutual information, Proportion of Ambiguous Clustering	Used to evaluate similarity of results obtained on different subsets of data
**Prognostic relevance**	**Likelihood-ratio-test of Cox proportional hazards models**, Harrell’s concordance-index	Used to evaluate the differential survival between clusters while accounting for covariates
**Clinical relevance**	ANOVA, Kruskal-Wallis, Chi-squared statistical tests, **Adjusted Rand Index**, Jaccard index and normalized mutual information	Used to assess associations to various patient clinical data, such as known subtypes or lifestyle

### Clustering stability

To evaluate clustering stability, we employed the Jaccard index within a repeated cross-validation (CV) framework. This method belongs in the category of subsampling stability methods which includes the more popular bootstrap. The benefit of CV is that the dataset is sampled evenly, allowing even coverage of the data in each repeat of CV while with bootstrap some points would be sampled significantly fewer times than the average. First, we obtained a reference clustering result from the full dataset. Next, we compared the clusters from each cross-validation training set iteration to this reference [55]. The more similar the clusters are the more stable the clustering algorithm is. Similarity was quantified using the Jaccard index, treating the comparison as binary classification applied to the connectivity matrices. In more detail, a 2x2 contingency table is formulated, detailing observation pairs as follows: those in belonging to the same cluster in both results (TP); same in the first but not the second (FP); same in the second but not the first (FN); and pairs belonging to different clusters in both (TN). Then, the Jaccard index can be framed in the following formula: TP /(TP+FP+FN). [28] Using a reference from full data allows all samples in a given fold to be used for comparison, while comparisons between folds would reduce the proportion of samples to (N_folds– 2) / N_folds due to the definition of CV. Bootstrapping with replacement should not be used since duplicated data points belong to the same cluster and would therefore incorrectly inflate the stability measurement by definition. More recent stability metrics such as the proportion of ambiguous clustering (PAC) [56] have been suggested as a metric for consensus clustering analysis, but it can be applied within any resampling based clustering analysis. PAC is included as an alternative metric in COPS, but it may require more resampling iterations to yield robust estimates since its resolution depends on the number of times each pair of points has been resampled together, while CV Jaccard avoids this problem by aggregating pairs across the subsample. Therefore, for computationally expensive methods, such as iCluster+ and MOFA, CV Jaccard is a more attractive metric.

### Survival analysis

Pre-processed survival data was retrieved from [57] where the authors updated and quality-checked the survival data available from TCGA cancer datasets. Depending on the aggressiveness of each cancer type, either the Overall survival (OS) or progression-free interval (PFI) was used (see Table 1). To account for covariates in survival analysis Cox proportional hazards (PH) models were used while assessing the differential survival between clusters. For each CV fold and clustering result, a baseline model was fitted with covariates as predictors and compared to an augmented model with the cluster indicators as additional predictors. To assess the significance a likelihood-ratio test was performed between the baseline and augmented models with the null hypothesis being that the cluster indicator coefficients are 0. The covariates were selected based on availability and included the age of the patient and AJCC stage assessed by [57] as well as patient sex where appropriate, i.e., sex was excluded from BRCA, PRAD and ovarian cancer covariates. We assessed the relevance of different covariates in each cancer by recursively adding variables to Cox models starting from a model with only intercept. In most cancers there was only one highly relevant covariate while ovarian and prostate cancer had none, possibly due to the lack of AJCC staging for these cancers in the dataset. S9 Fig shows the covariate assessment results in more detail.

### Association with known subtypes

We evaluated the quality of the resulting patient stratification by comparing it with known cancer subtypes. Since the matching of multi-class labels to clusters is ambiguous, we used the Adjusted Rand Index (ARI) score, which measures the similarity between two partitions (i.e., clusters and known subtypes) adjusted for chance [29], to measure the clustering accuracy.

### Pareto efficiency

In this study, we utilized a multi-objective optimization approach, minimizing some objectives while maximizing others. We used the Pareto optimal set of non-dominated solutions to gauge the best multi-view clustering algorithms. A solution is deemed Pareto optimal if no other solution outperforms it in any objective without being inferior in another. Given *m* objectives *f*_*1*_, *f*_*2*_,…, *f*_*m*_ to be minimized and *n* objectives *g*_*1*_, *g*_*2*_,…, *g*_*n*_ to be maximized, a solution *x* is said to dominate another solution *y* (denoted as *x ≺ y*) if *f*_*i*_*(x) ≤ f*_*i*_*(y)* for all *i* in *{1*, *2*,…, *m}*, *g*_*j*_*(x) ≥ g*_*j*_*(y)* for all *j* in *{1*, *2*,…, *n}*, and there is at least one *k* such that either *f*_*k*_*(x) < f*_*k*_*(y*) or *g*_*k*_*(x) > g*_*k*_*(y)*. Our goal was to find all such non-dominated solutions, which form the Pareto frontier. The first pareto frontier was used to identify the solution that performed optimally in terms of Pareto efficiency.

**KEY POINTS**

- COPS introduces an innovative computational framework designed to benchmark multi-view clustering algorithms. By employing a cross-fold validation approach, it ensures the accuracy and reliability of clustering results. This meticulous validation method equips researchers with robust evaluation metrics, such as the silhouette score, ARI, stability, and survival, making it indispensable for rigorous comparisons and analyses in the rapidly evolving field of multi-omics data analysis.
- COPS offers swift access to an unparalleled collection of cutting-edge clustering methods for both single- and multi-omic data. Notably, it includes a crucial set of algorithms based on pathway- or network-driven approaches, which were overlooked in previous reviews.
- COPS implements two novel pathway kernel approaches BWK and RWR-BWK. The former was able to yield good performance in many case-studies and offered an optimal trade-off in LGG.
- The study underscores the importance of multi-objective evaluation in multi-omics-driven cancer subtyping. By utilizing the Pareto optimal concept, the analysis seeks to balance and prioritize multiple clustering outcomes. In the context of cancer patient stratification, a Pareto optimal solution aims to find the best trade-offs among various metrics like survival, stability, silhouette score, and cluster cohesion, ensuring no single metric is optimized at the detriment of another, leading to the most comprehensive patient stratification results.

## Supporting information

S1 Fig Algorithms and evaluation metrics integrated in the COPS framework. COPS employs three primary steps for patient stratification: feature extraction, clustering, and the evaluation of clustering outcomes. Feature extraction can be executed for single-omics data types using data-driven methods like PCA, UMAP, and Similarity Network. For pathway-based transformation of omics data types, techniques such as GSVA, DiffRank, and RWR-GSEA are employed. A notable method, Multi-Omics Factor Analysis, facilitates the extraction of factors characterized by a set of various omics-based molecular features. The clustering process can be implemented for single-omics data types using conventional techniques such as Hierarchical Clustering, K-means, Gaussian Mixture Models, and Spectral Clustering. One of the key innovations presented in this tool is Multi-View Clustering, which includes both data-driven methods like iCluster, SNF, ANF, IntNMF, and Kernel-based methods. The latter has been expanded to encompass algorithms providing a pathway-based kernel transformation, such as PAMOGK, BWK, and PIK. COPS provides a variety of evaluation metrics, covering clustering separation metrics, association with known subtypes (e.g., assessed through the ARI score), and clinical relevance assessment (like survival with Cox regression models). It also allows to implement Pareto-based multi-objective evaluations for a comprehensive understanding of clustering results.
(TIFF)

S2 Fig COPS resampled clustering analysis with cross-fold validation. Each dataset is partitioned into five folds, and then training sets, derived by combining n-1 folds in a loop, are used to assess the clustering performance of all chosen methods and evaluation criteria. This procedure is replicated 10 times, yielding a total of 100 performance tests. Subsequently, the distribution of each metric for each dataset and clustering algorithm is harnessed to generate boxplots and to pinpoint the optimal solutions based on the Pareto-optimal criterion (or non-dominated solutions), also known as Pareto efficiency.
(TIFF)

S3 Fig Stability of cancer clustering results with different feature filters. The boxplots show Jaccard-index-based stability for different multi-omics clustering approaches and different number of clusters (k) across 10 repeats of 5-fold cross-validation. The box and middle line represent the second and third quartiles and the median while the whiskers extend to the maximum value or 1.5 times inter-quartile range from the box edges. The features were selected from gene-summarized omics by selecting the top M genes from each omic by variance and taking their union.
(TIFF)

S4 Fig Stability of cancer clustering results with different pathway sets. The boxplots show Jaccard-index-based stability for different multi-omics clustering approaches and different number of clusters (k) across 10 repeats of 5-fold cross-validation. The box and middle line represent the second and third quartiles and the median while the whiskers extend to the maximum value or 1.5 times inter-quartile range from the box edges. PWDB, pathway database; KEGG, Kyoto Encyclopedia of Genes and Genomes; PID, National Cancer Institute Pathway Interaction Database.
(TIFF)

S5 Fig Cluster survival results with different pathway sets. The boxplots show the p-value of a likelihood-ratio test between Cox PH models such that known covariates are accounted for. Values for different multi-omics clustering approaches and different number of clusters (k) across 10 repeats of 5-fold cross-validation. The box and middle line represent the second and third quartiles and the median while the whiskers extend to the maximum value, or 1.5 times inter-quartile range from the box edges. The red line represents the threshold p = 0.05. PWDB, pathway database; KEGG, Kyoto Encyclopedia of Genes and Genomes; PID, National Cancer Institute Pathway Interaction Database.
(TIFF)

S6 Fig Cancer subtype agreement for all numbers of clusters. The boxplots show the adjusted rand index (ARI) between the clusters and gold-standard subtypes on the y-axis for the considered multi-omics clustering approaches and different number of clusters (k) across 10 repeats of 5-fold cross-validation. The box and middle line represent the second and third quartiles and the median while the whiskers extend to the maximum value or 1.5 times inter-quartile range from the box edges.
(TIFF)

S7 Fig Cancer subtype agreement with different pathway sets. The boxplots show the adjusted rand index (ARI) between the clusters and gold-standard subtypes on the y-axis. For each dataset only the clustering result corresponding to the known number of subtypes is shown for the considered multi-omics clustering approaches across 10 repeats of 5-fold cross-validation. The box and middle line represent the second and third quartiles and the median while the whiskers extend to the maximum value or 1.5 times inter-quartile range from the box edges. PWDB, pathway database; KEGG, Kyoto Encyclopedia of Genes and Genomes; PID, National Cancer Institute Pathway Interaction Database.
(TIFF)

S8 Fig Size of the smallest cluster. Size as the fraction of points belonging to the smallest cluster compared to the size of the dataset on the y-axis for the considered multi-omics clustering approaches and different number of clusters (k) across 10 repeats of 5-fold cross-validation. The box and middle line represent the second and third quartiles and the median while the whiskers extend to the maximum value or 1.5 times inter-quartile range from the box edges.
(TIFF)

S9 Fig Survival covariate significance in survival prediction. The barplots show the p-value of a likelihood-ratio-test between Cox models that were fitted on different covariates. The color represents the null model that is compared against an augmented model with the covariate on the x-axis included in addition to the null model covariates. The null model with intercept-only is represented as “1”. Ovarian and prostate cancer were omitted due to missing covariates and insignificant results.
(TIFF)

S1 Table List of algorithms included in the COPS-package. Different types of methods are listed including biology- and data-driven feature extraction methods, single-view clustering methods as well as multi-omic algorithms. Some methods were imported from existing R-packages, while many were implemented from scratch in COPS.
(XLSX)

S2 Table Number of highest variance genes selected per omic for each dataset-approach combination. Each combination was tested with 500, 1000, and 2000 genes per omic and the number of genes was selected based on the average clustering performance of each dataset-approach combination.
(XLSX)
