# Supplementary figures and images for: COPS: A novel platform for multi-omic disease subtype discovery via robust multi-objective evaluation of clustering algorithms
Source: PLoS Comput Biol. 2024 Aug 5;20(8):e1012275. doi: 10.1371/journal.pcbi.1012275 (PMC11326705; doi:10.1371/journal.pcbi.1012275)

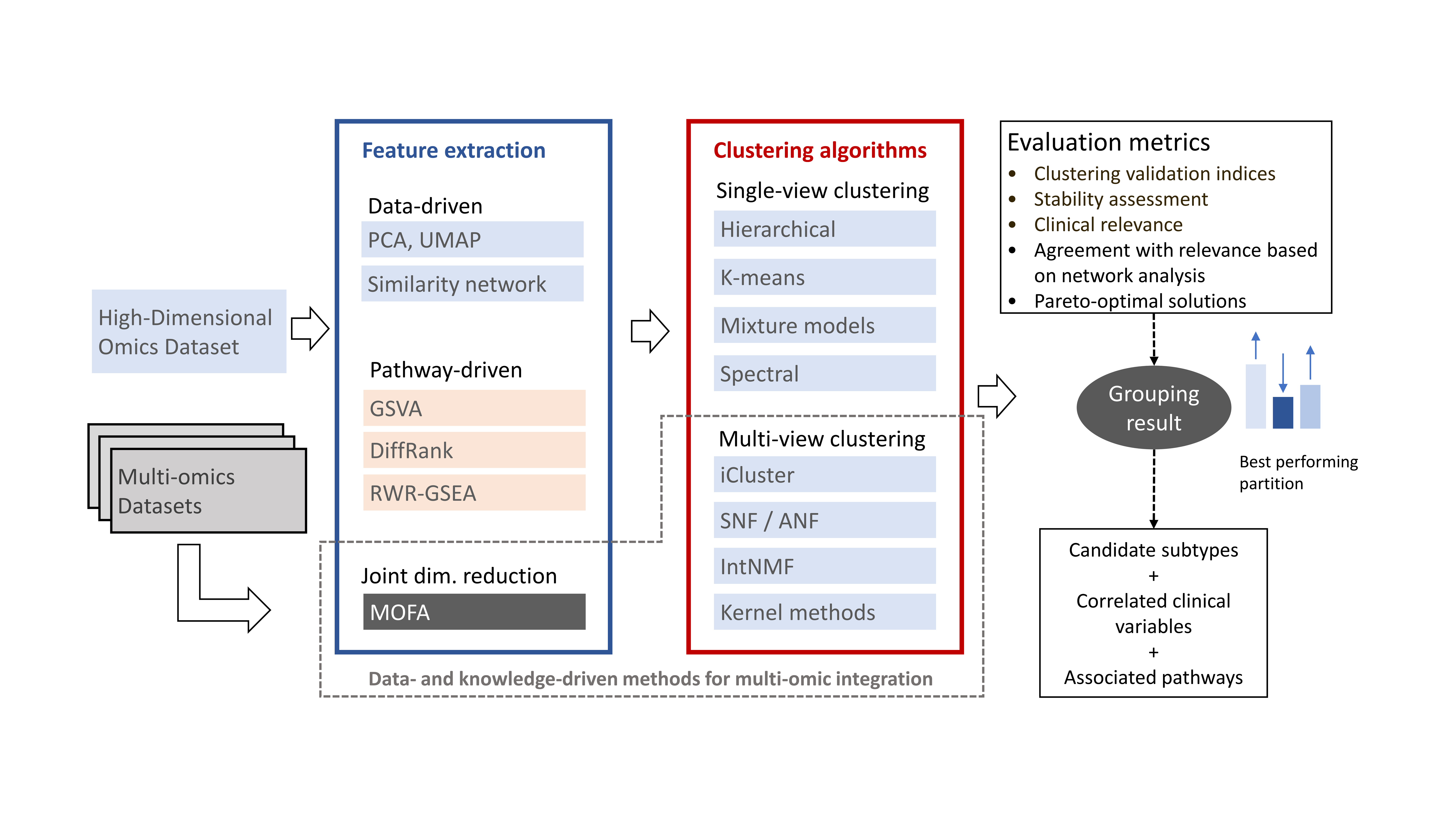

Supplement: S1 Fig — COPS employs three primary steps for patient stratification: feature extraction, clustering, and the evaluation of clustering outcomes. Feature extraction can be executed for single-omics data types using data-driven methods like PCA, UMAP, and Similarity Network. For pathway-based transformation of omics data types, techniques such as GSVA, DiffRank, and RWR-GSEA are employed. A notable method, Multi-Omics Factor Analysis, facilitates the extraction of factors characterized by a set of various omics-based molecular features. The clustering process can be implemented for single-omics data types using conventional techniques such as Hierarchical Clustering, K-means, Gaussian Mixture Models, and Spectral Clustering. One of the key innovations presented in this tool is Multi-View Clustering, which includes both data-driven methods like iCluster, SNF, ANF, IntNMF, and Kernel-based methods. The latter has been expanded to encompass algorithms providing a pathway-based kernel transformation, such as PAMOGK, BWK, and PIK. COPS provides a variety of evaluation metrics, covering clustering separation metrics, association with known subtypes (e.g., assessed through the ARI score), and clinical relevance assessment (like survival with Cox regression models). It also allows to implement Pareto-based multi-objective evaluations for a comprehensive understanding of clustering results. (TIFF) [file pcbi.1012275.s001.tiff]

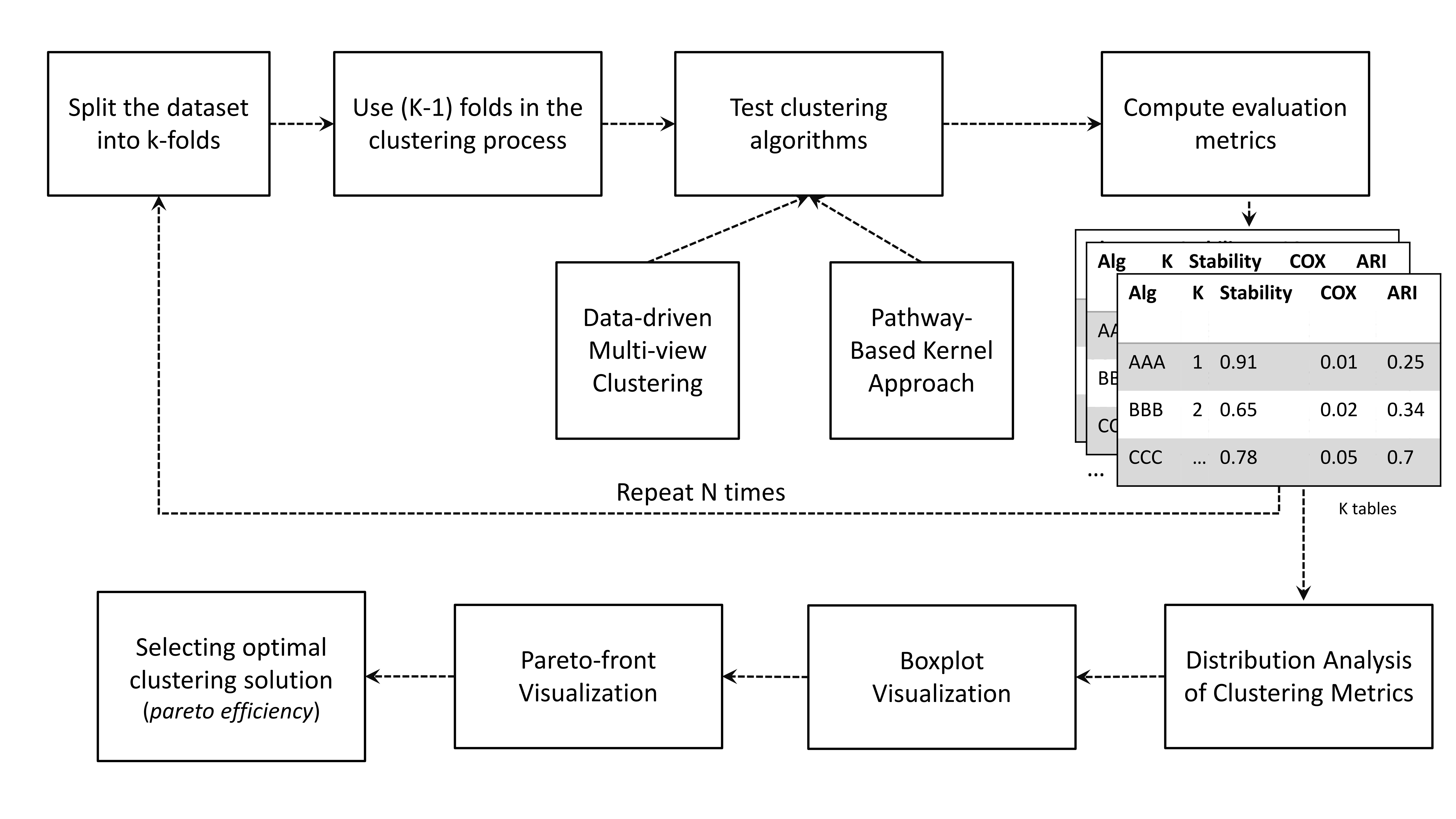

Supplement: S2 Fig — Each dataset is partitioned into five folds, and then training sets, derived by combining n-1 folds in a loop, are used to assess the clustering performance of all chosen methods and evaluation criteria. This procedure is replicated 10 times, yielding a total of 100 performance tests. Subsequently, the distribution of each metric for each dataset and clustering algorithm is harnessed to generate boxplots and to pinpoint the optimal solutions based on the Pareto-optimal criterion (or non-dominated solutions), also known as Pareto efficiency. (TIFF) [file pcbi.1012275.s002.tiff]

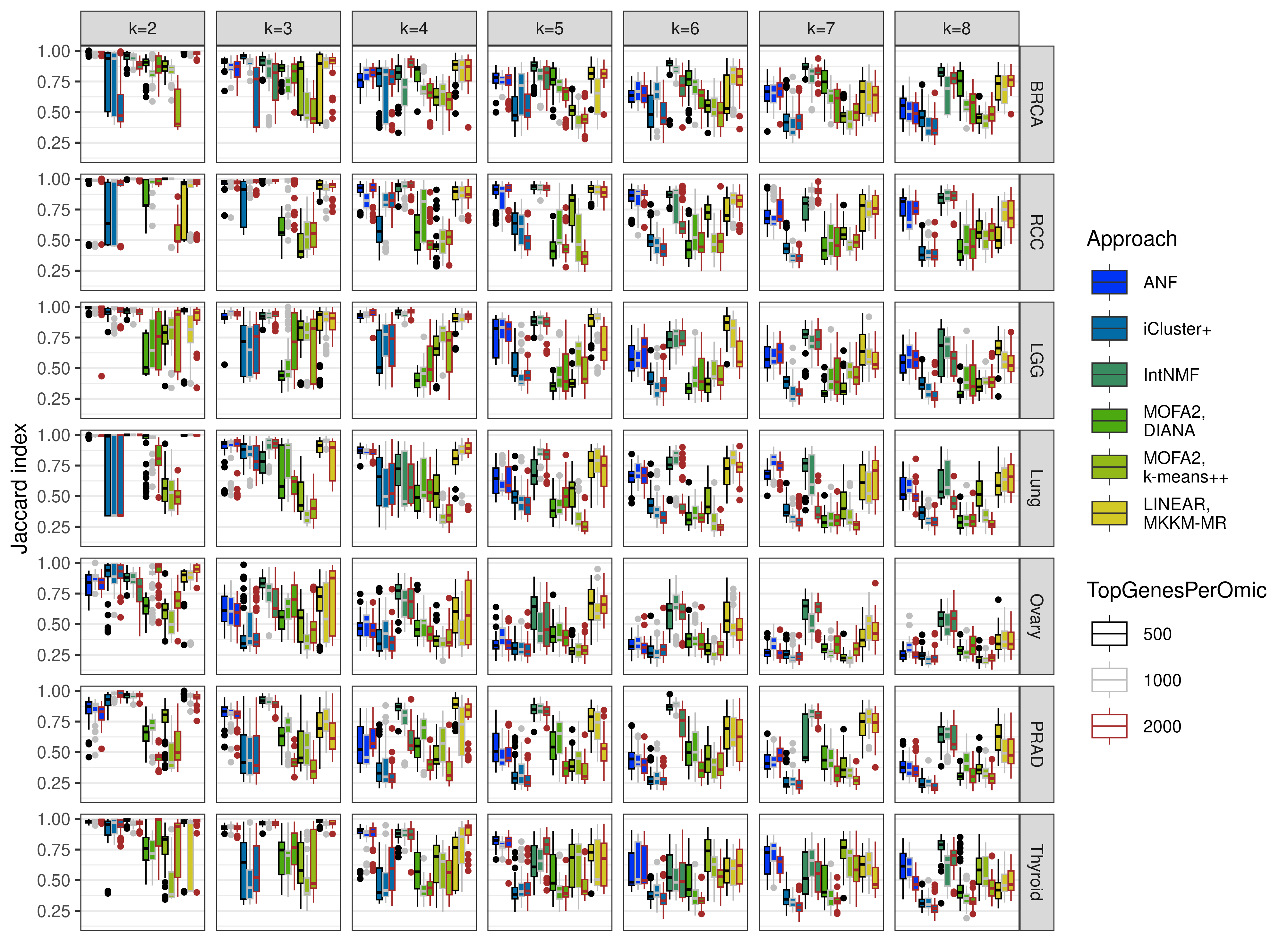

Supplement: S3 Fig — The boxplots show Jaccard-index-based stability for different multi-omics clustering approaches and different number of clusters (k) across 10 repeats of 5-fold cross-validation. The box and middle line represent the second and third quartiles and the median while the whiskers extend to the maximum value or 1.5 times inter-quartile range from the box edges. The features were selected from gene-summarized omics by selecting the top M genes from each omic by variance and taking their union. (TIFF) [file pcbi.1012275.s003.tiff]

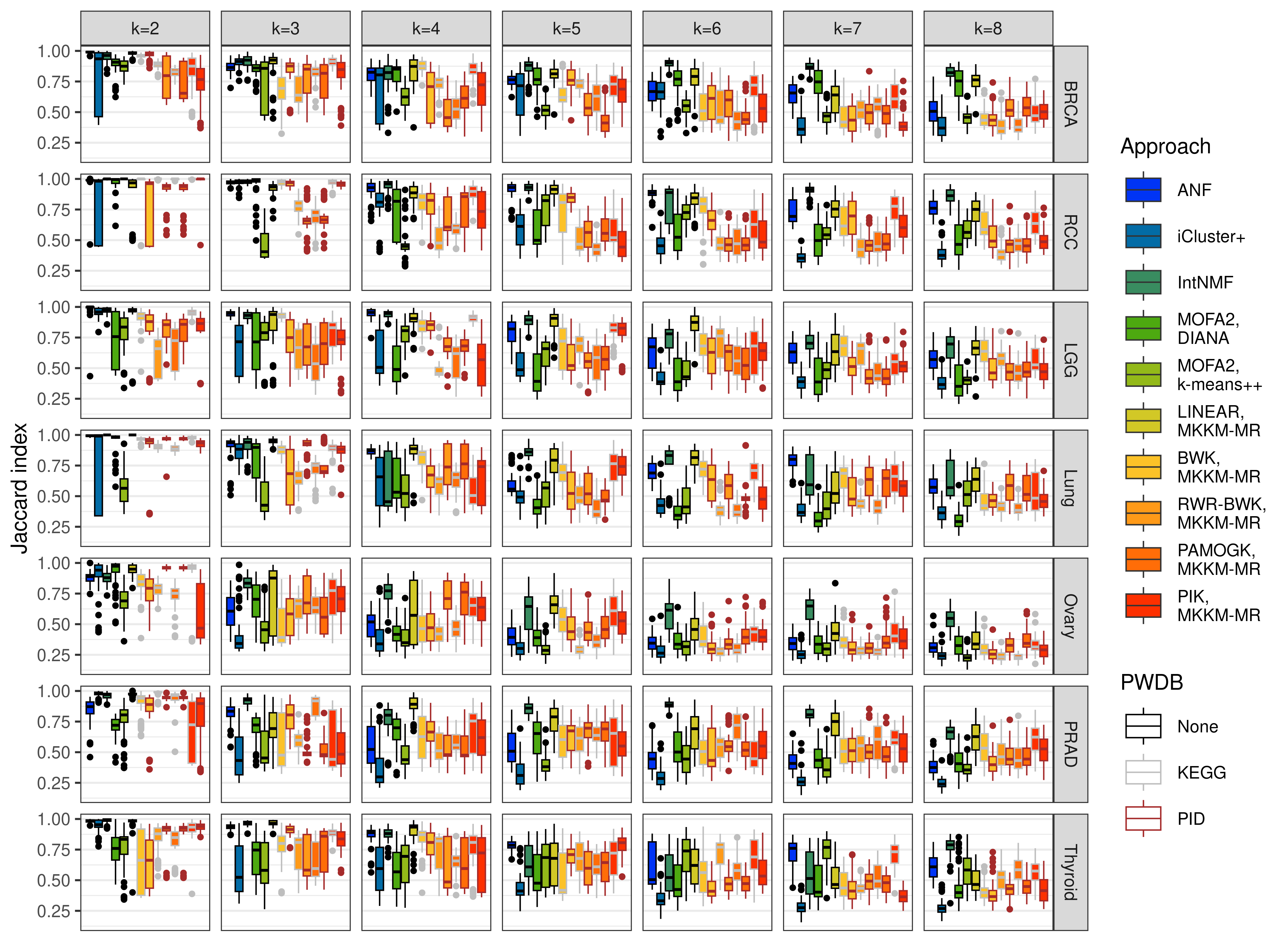

Supplement: S4 Fig — The boxplots show Jaccard-index-based stability for different multi-omics clustering approaches and different number of clusters (k) across 10 repeats of 5-fold cross-validation. The box and middle line represent the second and third quartiles and the median while the whiskers extend to the maximum value or 1.5 times inter-quartile range from the box edges. PWDB, pathway database; KEGG, Kyoto Encyclopedia of Genes and Genomes; PID, National Cancer Institute Pathway Interaction Database. (TIFF) [file pcbi.1012275.s004.tiff]

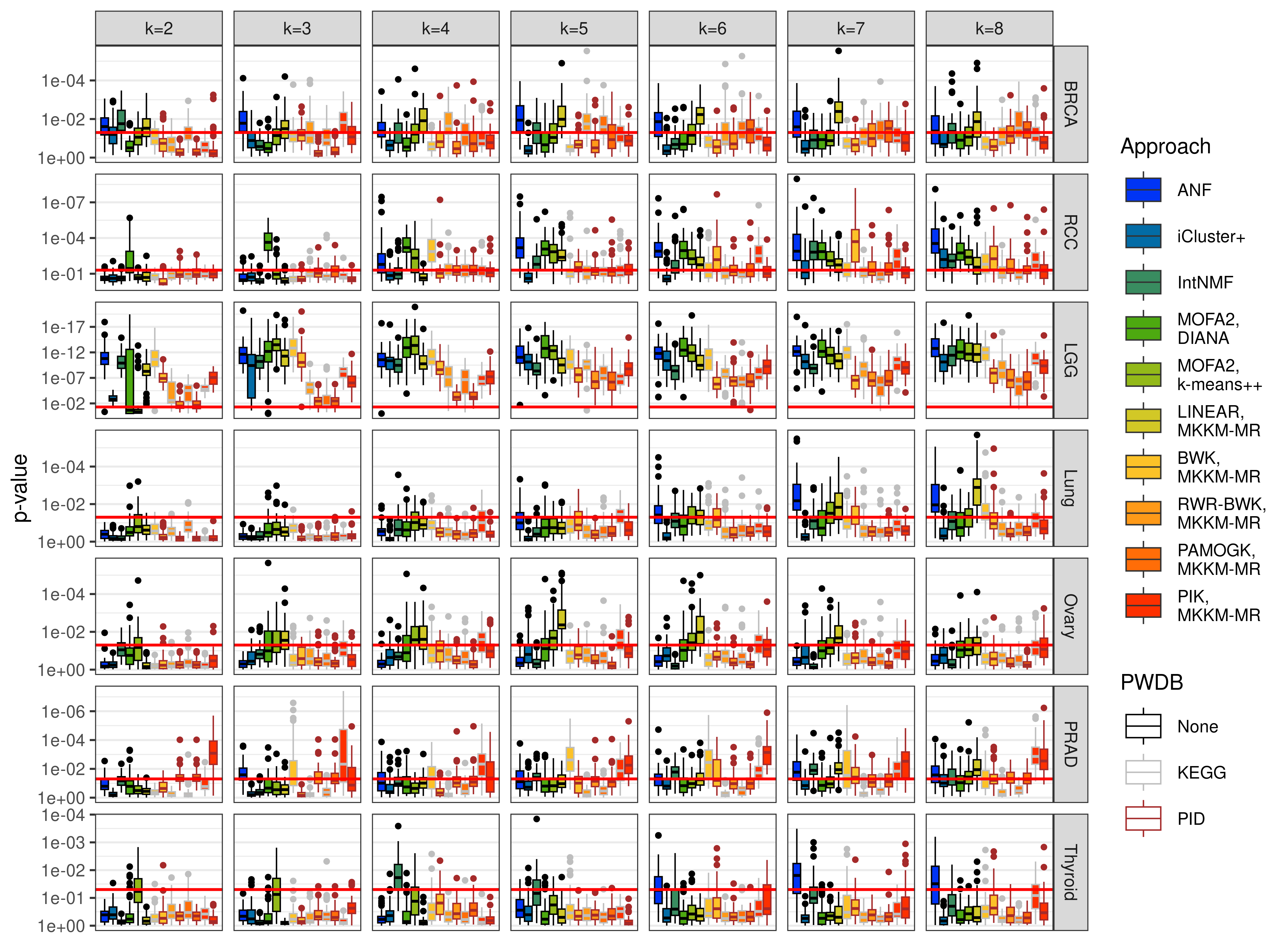

Supplement: S5 Fig — The boxplots show the p-value of a likelihood-ratio test between Cox PH models such that known covariates are accounted for. Values for different multi-omics clustering approaches and different number of clusters (k) across 10 repeats of 5-fold cross-validation. The box and middle line represent the second and third quartiles and the median while the whiskers extend to the maximum value, or 1.5 times inter-quartile range from the box edges. The red line represents the threshold p = 0.05. PWDB, pathway database; KEGG, Kyoto Encyclopedia of Genes and Genomes; PID, National Cancer Institute Pathway Interaction Database. (TIFF) [file pcbi.1012275.s005.tiff]

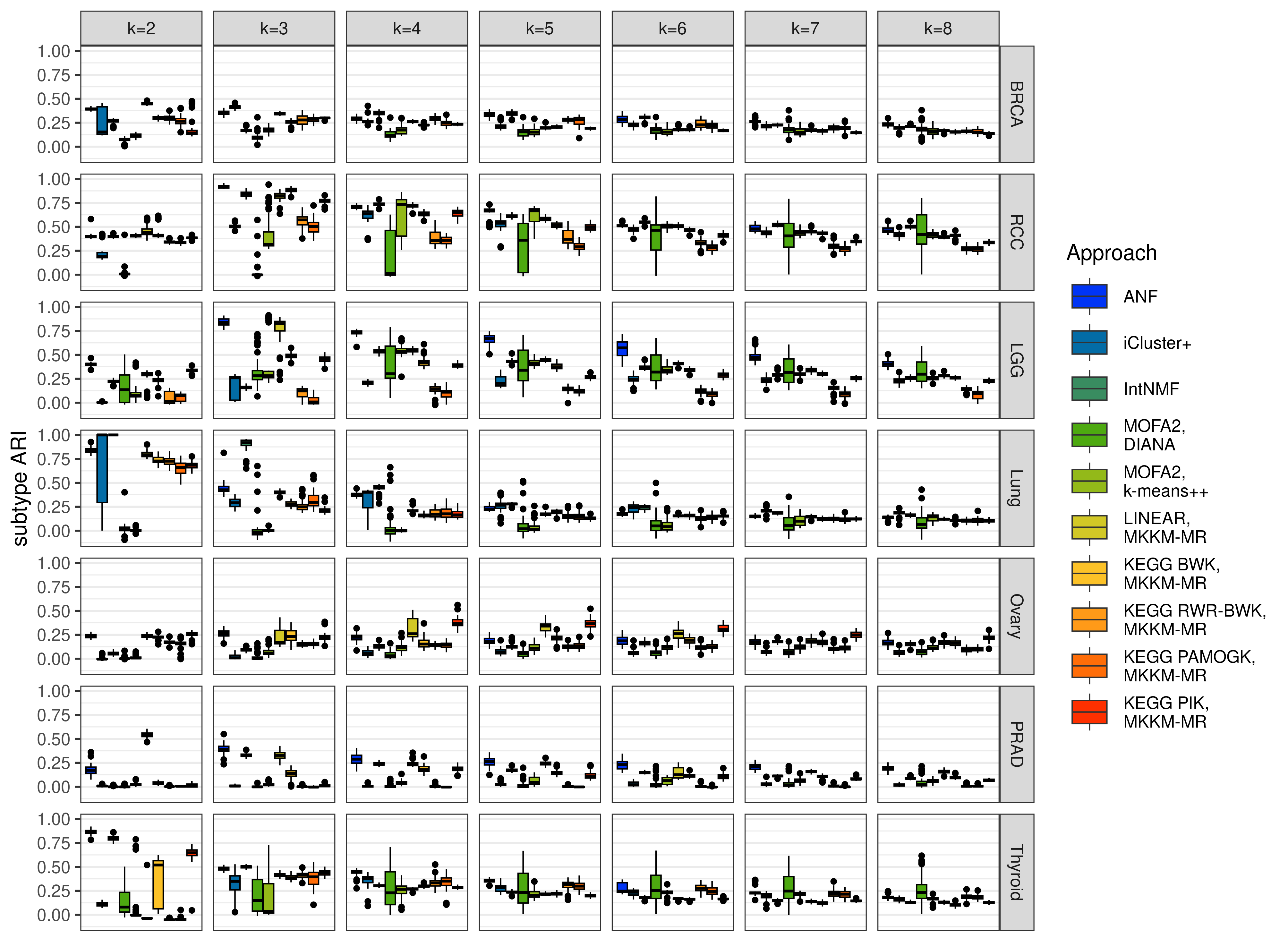

Supplement: S6 Fig — The boxplots show the adjusted rand index (ARI) between the clusters and gold-standard subtypes on the y-axis for the considered multi-omics clustering approaches and different number of clusters (k) across 10 repeats of 5-fold cross-validation. The box and middle line represent the second and third quartiles and the median while the whiskers extend to the maximum value or 1.5 times inter-quartile range from the box edges. (TIFF) [file pcbi.1012275.s006.tiff]

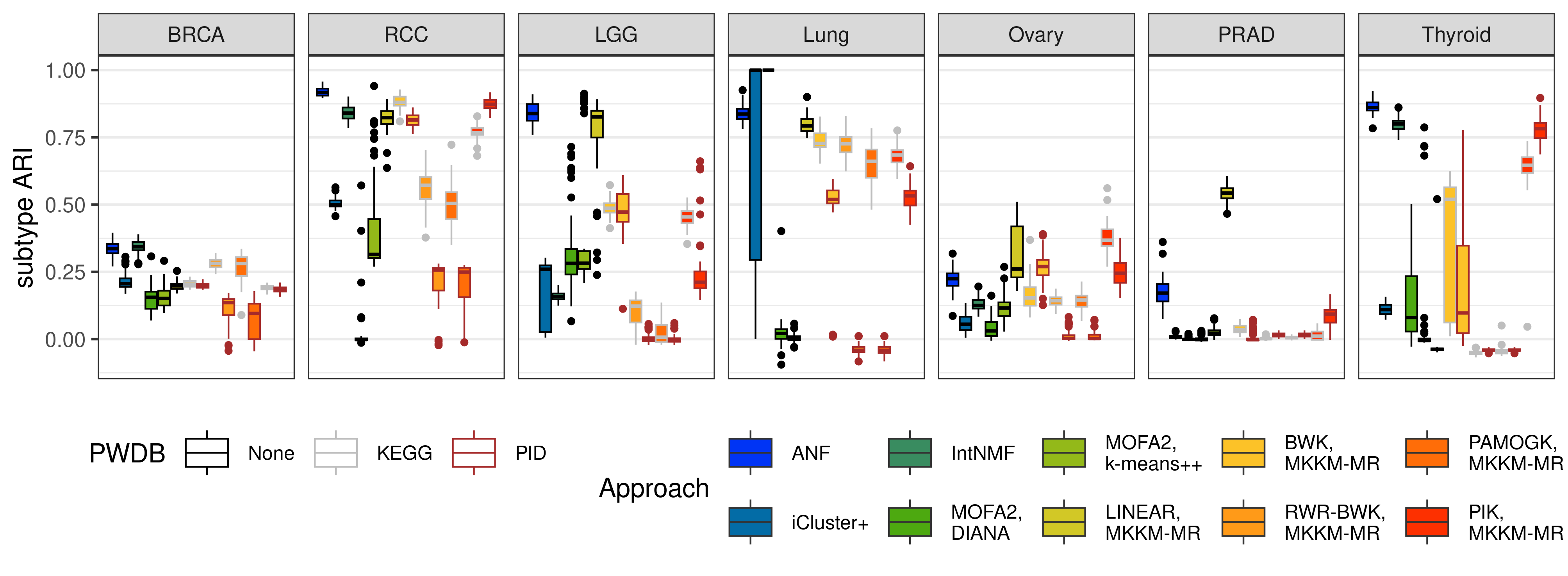

Supplement: S7 Fig — The boxplots show the adjusted rand index (ARI) between the clusters and gold-standard subtypes on the y-axis. For each dataset only the clustering result corresponding to the known number of subtypes is shown for the considered multi-omics clustering approaches across 10 repeats of 5-fold cross-validation. The box and middle line represent the second and third quartiles and the median while the whiskers extend to the maximum value or 1.5 times inter-quartile range from the box edges. PWDB, pathway database; KEGG, Kyoto Encyclopedia of Genes and Genomes; PID, National Cancer Institute Pathway Interaction Database. (TIFF) [file pcbi.1012275.s007.tiff]

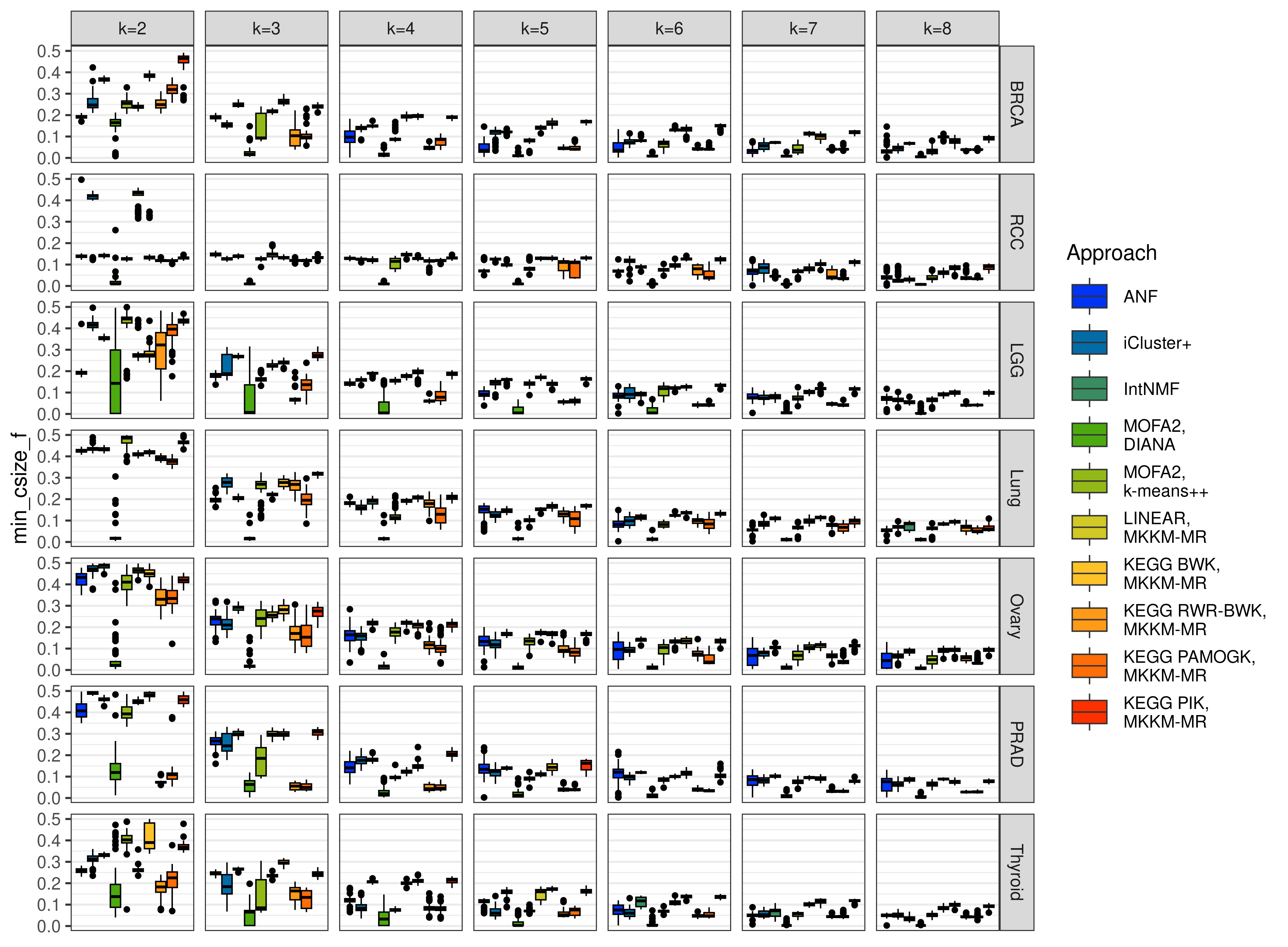

Supplement: S8 Fig — Size as the fraction of points belonging to the smallest cluster compared to the size of the dataset on the y-axis for the considered multi-omics clustering approaches and different number of clusters (k) across 10 repeats of 5-fold cross-validation. The box and middle line represent the second and third quartiles and the median while the whiskers extend to the maximum value or 1.5 times inter-quartile range from the box edges. (TIFF) [file pcbi.1012275.s008.tiff]

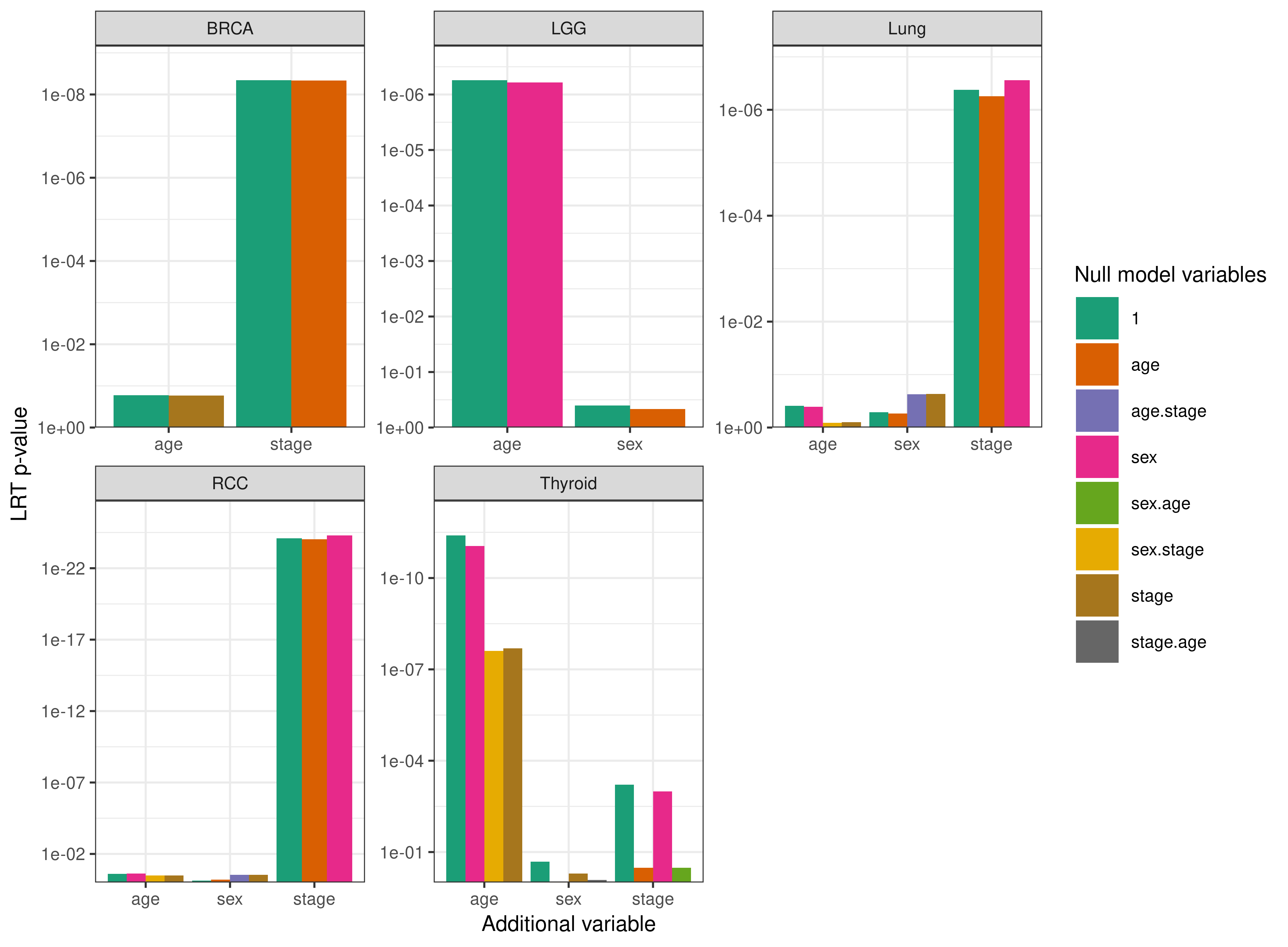

Supplement: S9 Fig — The barplots show the p-value of a likelihood-ratio-test between Cox models that were fitted on different covariates. The color represents the null model that is compared against an augmented model with the covariate on the x-axis included in addition to the null model covariates. The null model with intercept-only is represented as “1”. Ovarian and prostate cancer were omitted due to missing covariates and insignificant results. (TIFF) [file pcbi.1012275.s009.tiff]
